# Co-exposure to benzo[a]pyrene and ethanol induces a pathological progression of liver steatosis *in vitro* and *in vivo*

**DOI:** 10.1038/s41598-018-24403-1

**Published:** 2018-04-13

**Authors:** Simon Bucher, Arnaud Tête, Normand Podechard, Marie Liamin, Dounia Le Guillou, Martine Chevanne, Cédric Coulouarn, Muhammad Imran, Isabelle Gallais, Morgane Fernier, Quentin Hamdaoui, Marie-Anne Robin, Odile Sergent, Bernard Fromenty, Dominique Lagadic-Gossmann

**Affiliations:** 1 Univ Rennes, Inserm, Inra, Institut NUMECAN (Nutrition Metabolisms and Cancer) - UMR_S 1241, UMR_A 1341, F-35000 Rennes, France; 2 Univ Rennes, Inserm, EHESP, Irset (Institut de recherche en santé, environnement et travail) - UMR_S 1085, F-35000 Rennes, France

## Abstract

Hepatic steatosis (i.e. lipid accumulation) and steatohepatitis have been related to diverse etiologic factors, including alcohol, obesity, environmental pollutants. However, no study has so far analyzed how these different factors might interplay regarding the progression of liver diseases. The impact of the co-exposure to the environmental carcinogen benzo[a]pyrene (B[a]P) and the lifestyle-related hepatotoxicant ethanol, was thus tested on *in vitro* models of steatosis (human HepaRG cell line; hybrid human/rat WIF-B9 cell line), and on an *in vivo* model (obese zebrafish larvae). Steatosis was induced prior to chronic treatments (14, 5 or 7 days for HepaRG, WIF-B9 or zebrafish, respectively). Toxicity and inflammation were analyzed in all models; the impact of steatosis and ethanol towards B[a]P metabolism was studied in HepaRG cells. Cytotoxicity and expression of inflammation markers upon co-exposure were increased in all steatotic models, compared to non steatotic counterparts. A change of B[a]P metabolism with a decrease in detoxification was detected in HepaRG cells under these conditions. A prior steatosis therefore enhanced the toxicity of B[a]P/ethanol co-exposure *in vitro* and *in vivo*; such a co-exposure might favor the appearance of a steatohepatitis-like state, with the development of inflammation. These deleterious effects could be partly explained by B[a]P metabolism alterations.

## Introduction

Hepatic steatosis, or fatty liver disease, is a growing epidemic characterized by an accumulation of lipids (mainly triglycerides) in hepatocytes. Although steatosis has long been considered as a benign liver disease, this state renders the liver more susceptible to further harmful stress, then leading to chronic cell death and inflammation, the so-called steatohepatitis^1,2^. This chronic inflammatory state forms the fertile ground for more severe liver diseases, namely fibrosis, cirrhosis and cancer^3,4^. When unrelated to alcohol, both steatosis and steatohepatitis are generally gathered under the term NAFLD for nonalcoholic fatty liver disease, with steatohepatitis termed as NASH for nonalcoholic steatohepatitis^1,2^. NAFLD currently affects around 30% of worldwide general population, and is considered as the most common chronic liver disease in several countries, particularly in high-fat diet (HFD)-consuming countries^1,2,5,6^. As obesity predisposes in most cases to steatosis and due to the increasing prevalence of obesity, a further increase in NAFLD is expected in the near future with even more serious consequences in terms of clinics and health costs^1,7^. NAFLD therefore constitutes a major public concern and thus deserves more thorough investigation, notably regarding the factors favoring the pathologic progression of steatosis towards steatohepatitis.

Although fatty liver and steatohepatitis most commonly stem from overnutrition and lack of exercise, other causes have been recently put forward, such as environmental factors. Indeed, several environmental toxicants, more recently termed metabolism-disrupting chemicals^8^, have been reported as perturbing the function of endocrine and metabolic organs, including the liver, a key controller of body lipid metabolism^9^. Although several of these chemicals could be obesogen^9,10^, not all would lead to an increase in body fat mass and insulin resistance. This is in this context that the terms of toxicant-associated fatty liver disease (TAFLD) and toxicant-associated steatohepatitis (TASH) have been proposed by Cave’s group, to indicate the spectrum of fatty liver injury in chemically exposed-non obese individuals^11–13^. Hence, hepatic steatosis and steatohepatitis can be caused by multiple etiologic factors, the three most frequent causes therefore being alcohol (alcoholic liver disease or ALD), obesity/metabolic syndrome, and environmental toxicants (including drugs), as recently reviewed^13^. These three major etiologies appear to exhibit differences as well as common pathways in terms of the mechanisms involved in the development of steatohepatitis^13^. In this context, how they could interplay remains an underexplored field, despite the fact that some reports indicate worsening of steatohepatitis when present in binary combination (alcohol and obesity^14,15^; environmental contaminants and obesity^9,16^; drugs and obesity^17^).

Therefore, the present study aimed at evaluating how these three different factors might interplay with respect to the progression of liver diseases. To do so, we decided to test the impact of the co-exposure to both the environmental carcinogen benzo[a]pyrene (B[a]P) and the lifestyle-related hepatotoxicant ethanol, following prior establishment of hepatic steatosis induced by either fatty acid (FA) supplementation (*in vitro*) or high fat diet (HFD; *in vivo*). The polycyclic aromatic hydrocarbon B[a]P is present in cigarette smoke, diesel exhaust particles as well as smoked and grilled food among others. In non-smokers, exposure occurs mainly *via* diet^18^. This well-recognized genotoxic carcinogen to humans is thus metabolized by the liver (see eg.^19^), and has been suggested to induce liver steatosis^20,21^ as well as hepatocellular carcinoma (HCC), especially in human^22,23^. Besides, epidemiological studies suggest a synergistic effect of B[a]P and alcohol on HCC risk^24^. Moreover, we recently evidenced a cooperative interaction of B[a]P and ethanol towards cell death in rat primary hepatocytes^25^. In this context, we decided to work on several biological models of hepatic steatosis in order to get strong support regarding our findings. First, we used the human HepaRG cell line since this is physiologically one of the closest cell lines to primary human hepatocyte^26^. Secondly, the hybrid human/rat WIF-B9 cell line was chosen due to its high level of differentiation into hepatocyte and its sensitivity to low concentrations of chemicals, notably alcohol^27,28^, compared to HepaRG cells; such a feature appears to be interesting when studying concentrations of chemicals relevant to human exposure. Finally, we focused our study on the zebrafish larva model to test *in vivo* our hypothesis; indeed this model is now well recognized as sharing pathophysiological processes with human, especially concerning liver diseases, with advantages of time and cost-efficiency in comparison to mammal or rodent models^29–31^.

The present study showed for the first time that the presence of a prior steatosis enhanced the toxicity of B[a]P/ethanol co-exposure both *in vitro* and *in vivo*, and that such a co-exposure, even at sub-toxic concentrations, might favor the appearance of a steatohepatitis-like state with an increased expression of several inflammation markers. Alterations in xenobiotic metabolism may explain, at least in part, some of these deleterious effects.

## Methods

### *In vitro* and *in vivo* models of liver steatosis

For both cell line models, phases of steatosis induction and B[a]P/ethanol treatments were determined to be an optimal compromise between a proper differentiated hepatocyte state and a maximum duration of treatment that cells could undergo. Protocols of exposure for all models are given in Fig. S1.

#### HepaRG cell culture and treatments

HepaRG cells were cultured according to the standard protocol previously described^32^. After 2 weeks, cell differentiation was induced with 2% DMSO for 2 additional weeks. Differentiated cells were then treated during 16 days with or without a mixture of fatty acids (150 µM stearic acid and 150 µM oleic acid; see supplementary Methods for commercial source, and Fig. S1 for exposure protocol) in a medium containing 5% FBS and 1% DMSO. Our protocol of steatosis induction was adapted from a previous study carried out in HepaRG cells, for which both fatty acids were used for a 1-week period^33^. After 2 days from the onset of the experiments, steatotic and non-steatotic cells were treated with or without B[a]P and/or ethanol every 2 or 3 days. For cytotoxicity studies, B[a]P concentrations ranged from 0.01 to 50 µM, and ethanol concentrations were set to 25 and 50 mM. For all further experiments, the selected concentrations were 1 and 2.5 µM for B[a]P and 25 mM for ethanol.

#### WIF-B9 cell culture and treatments

WIF-B9 is a hybrid cell line obtained by fusion of Fao rat hepatoma cells and WI-38 human fibroblasts^34^. The WIF-B9 cells were a generous gift from Dr Doris Cassio (UMR Inserm S757, Université Paris-Sud, Orsay, France). Cells were cultured in F-12 Ham medium with Coon’s modification containing 5% FCS, 0.22 g/L sodium bicarbonate, 100 U/mL penicillin, 0.1 mg/mL streptomycin, 0.25 μg/mL amphotericin B, 2 mM glutamine, and supplemented with HAT (10 μM hypoxanthine, 40 nM aminopterin, 1.6 μM thymidine). WIF-B9 cells were seeded at 12.5 × 10^3^ cells/cm^2^; cells were cultured for 7 days until obtaining ∼80% of confluence, before treatment.

The FA-albumin complex containing medium was prepared by FA saponification with a NaOH/ethanol solution at 70 °C for 30 min. After ethanol evaporation under nitrogen, FA salts were solubilized in culture medium supplemented with 90 µM FA-free bovine serum albumin. The FA/albumin molar ratio was 6.1:1. Steatosis was induced by a two days treatment with a medium containing the FA/albumin complex composed of 450 µM oleic acid and 100 µM palmitic acid. Steatotic and non-steatotic cells were then exposed or not for an overall 5 days period to the toxicants (10 nM B[a]P with or without 5 mM ethanol; see Fig. S1 for exposure protocol). Media and treatments with toxicants were renewed on day 3 and kept until end of experiment. Regarding the time of xenobiotic exposure for these cells, the choice of 5 days was based on previous data showing that for longer treatments of non-steatotic cells with B[a]P, there might be a compensatory proliferation (unpublished data).

#### Zebrafish larvae handling and exposures

Animals were handled, treated and killed in agreement with the European Union regulations concerning the use and protection of experimental animals (Directive 2010/63/EU). All protocols were approved by local ethic committee CREEA (Comité Rennais d’Ethique en matière d’Expérimentation Animale). Zebrafish fertilized embryos, collected following natural spawning, were obtained from the Structure Fédérative de Recherche Biosit (INRA LPGP, Rennes, France). Embryos and larvae were raised at 28 °C according to standard procedures. Zebrafish larvae (sex unknown) were maintained as previously described^35^. From 4 days post-fertilization (dpf) until last day of treatment renewal (at  9 dpf), larvae were fed daily during 1 hour before medium renewal with a standard diet (SD, 10% of fat, Tetramin®) or with a high fat diet (HFD) made of chicken egg yolk (53% of fat, Sigma-Aldrich). These diets were chosen in accordance with other publications particularly concerning the lack of standardized diet for zebrafish^36,37^. At 5 dpf, zebrafish larvae were treated by 43 mM ethanol directly added to the incubation medium and/or by 25 nM B[a]P in DMSO (DMSO final proportion: 0.001% v/v), or by this vehicle only (see Fig. S1 for exposure protocol).

### Evaluation of steatosis

#### Oil red O staining

WIF-B9 cells: Oil red O staining was performed to visualize neutral lipid droplet accumulation. Cells were washed in phosphate buffer saline (PBS), then stained for 10 min with a solution of 0.15% oil red O in 60% isopropanol-PBS. Staining was completed by the addition of hematoxylin and eosin for 1 min followed by two washes in PBS. Cell pictures were acquired using a Zeiss Axiolab microscope (Carl Zeiss Microscopy GmbH, Jena, Germany).

Zebrafish larvae: At 5 or 12 dpf, larvae were washed in PBS and then fixed in 4% paraformaldehyde in PBS at 4 °C for at least 12 h before being stained overnight in a solution of 0.15% oil red O in 60% isopropanol-PBS. Then, larvae were washed three times in PBS and mounted in 80% glycerol-PBS. Images of zebrafish larvae were acquired with a LEICA binocular loupe (LEICA Microsystems SAS, Nanterre, France) (magnification x40). Liver and larvae sizes were determined from these images using Fiji imaging processing software (ImageJ, National Institutes of Health, Bethesda, MD).

#### Triglyceride assays

HepaRG cells: Cellular triglyceride content was measured using a colorimetric kit purchased from Biovision (Milpitas, CA), using the manufacturer’s recommendations. The amount of cellular triglycerides was normalized to total proteins determined by the bicinchoninic acid (BCA) method.

WIF-B9 cells and zebrafish larvae: For both cell and larvae samples, total lipid extraction was performed according to the Folch method. Total lipids were dissolved in 50 µL of ethanol and 6 µL were used for triglyceride measurement with the LabAssay™ Triglyceride Kit (Wako Chemicals GmbH, Neuss, Germany), according to the manufacturer’s instructions. Briefly, 300 µL of reaction mix were added to each sample for 5 min at 37 °C, and absorbance at 600 nm and 700 nm was measured using a Spectrostar Nano microplate reader (BMG Labtech, Ortenberg, Germany). Finally, triglyceride concentration was determined after normalization of absorbance (Δ absorbance (abs) = abs at 600 nm minus abs 700 nm), and using a standard curve.

#### Cholesterol and free fatty acid assays in WIF-B9 cells

Total cholesterol and free fatty acids (FFAs) were also measured in steatotic and non-steatotic WIF-B9 cells after two days of treatment with the FA mixture or not. Cholesterol quantification was performed by the Infinity cholesterol kit (Thermo Fisher Scientific, Cergy Pontoise, France), according to the manufacturer’s instructions. Briefly, 200 µL of reaction solution was added to each sample for 30 minutes at 37 °C, and absorbance at 492 nm was then measured using a Spectrostar Nano microplate reader. Regarding FFA quantification, it was performed by the NEFA-HR kit (Wako Chemicals GmbH, Neuss, Germany) according to the manufacturer’s instructions. After addition of the reaction solution and incubation at 37 °C, absorbance at 546 nm and 660 nm was measured using a Spectrostar Nano microplate reader. FFA concentration was determined after normalization (Δ abs = abs at 546 nm minus abs at 660 nm).

### In vitro and *in vivo* toxicity assays

#### ATP levels and MTT test

ATP levels were measured with the CellTiter-Glo^®^ Luminescent Cell Viability assay purchased from Promega (Charbonnières, France), according to the manufacturer’s instructions. Luminescence was measured using the POLARstar Omega microplate reader (BMG Labtech, Ortenberg, Germany) or the Spectramax Gemini XS microplate spectrofluorometer (Molecular Devices, Sunnyvale, CA). For the MTT test, cells were rinsed with PBS and incubated during 1 hour with a MTT solution (0.5 mg/mL in a serum-free and DMSO-free medium). After washing, cells were lysed with pure DMSO. Absorbance at 540 nm was measured using the POLARstar Omega microplate reader.

#### Hoechst/sytox green staining

Apoptotic cell death in WIF-B9 cells was assessed by visualization of chromatin condensation or fragmentation after nuclear staining. After treatments, cells were stained with 50 μg/mL Hoechst 33342 and 93.5 nM Sytox green in the dark for 30 min at 37 °C. Cells were then examined by fluorescence microscopy using the ZEISS Axio Scope A1 microscope (>300 cells analyzed per condition of treatment).

#### Histological analysis of liver toxicity in zebrafish larvae

Histological analysis was performed as previously described^35^. Briefly, after treatments, larvae at 12 dpf were washed in PBS and then fixed in 4% paraformaldehyde in PBS at 4 °C before being embedded in paraffin. Then, 4 µm-sections were stained with hematoxylin, eosin and safran red (HES) and imaged on Nanozoomer NDP (Hamamatsu Photonics K.K., Japan) (magnification x400). Histological count of dead/damaged cells was performed from images (1 or 2 sections) of at least 3 larvae per condition. Damaged/dead cells were counted as cellular dropouts, ballooning or vacuolated hepatocytes.

### Analysis of gene mRNA expression

#### HepaRG cells

Total RNA was extracted from ∼10^6^ HepaRG cells with the Nucleospin^®^ RNA isolation system (Macherey-Nagel, Hoerdt, France), which included a DNase treatment step. RNA was then reverse-transcribed into cDNA using the High-Capacity cDNA Reverse Transcriptase kit (Thermo Fisher Scientific, Cergy Pontoise, France). Real-time quantitative PCR (RT-qPCR) was performed using the SYBR Green PCR Master Mix on an Applied Biosystems 7900HT Fast Real-Time PCR System (Applied Biosystem, Woolston, UK). Expression of the human TATA box binding protein (TBP) was used as reference, and the 2^−ΔΔCt^ method was used to express the relative expression of each selected gene. Sequences of the tested human primers are provided in Table S1. For the transcriptomic analysis in HepaRG cells, see supplementary Methods.

#### WIF-B9 cells

Total RNA was extracted from ∼10^6^ WIF-B9 cells with TRIzol^®^ reagent (Invitrogen, Cergy Pontoise, France) according to the manufacturer’s protocol. For each RNA sample, one µg of RNA was reverse-transcribed into cDNA using the High capacity cDNA Reverse Transcription Kit (Applied Biosystems). RT-qPCR was then performed using SYBR Green on the CFX384 Touch™ Real-Time PCR Detection System (Bio-Rad, Hercules, CA). Expression of the rat *β-actin* was used as reference, and the 2^−ΔΔCt^ method was used to express the relative expression of each selected gene. Sequences of the rat primers are provided in Table S1.

#### Zebrafish larvae

For RNA extraction, 10 to 20 larvae were pooled and homogenized in 100 µL PBS and total RNA was extracted with TRIzol® reagent according to the manufacturer’s protocol. RNA samples (1 μg) were then reverse-transcribed using the High capacity cDNA Reverse Transcription Kit. RT-qPCR (5 ng of cDNA per well) was performed using the same protocol as for the WIF-B9 cells. mRNA expression was normalized by means of *actb2, 18s* and *gapdh* mRNA levels. The 2^−ΔΔCt^ method was used to express the relative expression of each selected gene. Sequences of the zebrafish primers are provided in Table S1. For the evaluation of the hepatic mRNA expression of C-reactive protein (*crp)*, see supplementary Methods.

### Interleukin-6 quantification

The concentrations of interleukin-6 (IL-6) secreted by HepaRG cells in culture medium were measured using the Duoset ELISA kit (R&D Systems, Abingdon, United Kingdom), according to the manufacturer’s instructions. IL-6 concentration in each well was normalized by the amount of total proteins determined by the BCA method.

### Cytochrome P450 activity and HPLC analysis in HepaRG cells

#### Cytochrome P450 (CYP) activity

Cytochrome P450 2E1 (CYP2E1) activity was assessed by determining the formation of 6-hydroxychlorzoxazone (6-OH-CZX), as recently reported^33^. Ethoxyresorufin O-deethylase (EROD) activity was used to measure CYP1A1, CYP1A2 and CYP1B1 activities^38,39^ in HepaRG cells after the 14-day exposure with B[a]P and/or EtOH in steatotic or non steatotic cells. Resorufin formation was monitored using a POLARstar Omega microplate reader (BMG Labtech, Ortenberg, Germany); excitation and emission wavelengths were 520 and 590 nm, respectively. Reaction rates were determined under linear conditions and normalized to total protein concentrations.

#### B[a]P metabolite detection by HPLC

At the end of the 14-day treatments, cells were washed with warm PBS and incubated during 15 min in a red phenol-free William’s E medium at 37 °C and 5% of CO_2_. This step aimed at removing all B[a]P metabolites synthesized during the 14-day treatment. Next, the medium was replaced by a phenol red-free William’s E medium containing 25 µM B[a]P with or without 5 mM salicylamide, a strong inhibitor of phase II xenobiotic metabolism enzymes (XMEs). After 6 hours at 37 °C and 5% of CO_2_, the medium was collected and centrifuged 15 min at 20,000 g at 4 °C, and 50 µL of the supernatant was directly injected into the HPLC system. The HPLC analysis was performed with the Agilent 1100TM system equipped with an Accucore PFP column (150 mm × 3 mm, particle size 2.6 µm) coupled with a fluorescent detector, as used by others for B[a]P metabolite detection^40–42^. A gradient of 0.1% acetic acid and acetonitrile was used throughout the experiment at a flow rate of 0.650 mL/min. Acetonitrile proportion ranged from 12.5 to 50% for 25 min and from 50 to 90% for 1.5 min. The wavelengths used to detect B[a]P metabolites, including B[a]P trans-7,8-dihydrodiol and 3-OH-B[a]P-glucuronide, were 365 and 405 nm for excitation and emission, respectively. The peaks of the different B[a]P metabolites were identified comparing the spectra of the cells incubated with and without B[a]P. Metabolite levels were semi-quantified using the area of each peak compared to the control condition, and were normalized by the amount of proteins. The results were expressed as percentage of control values. The peaks of B[a]P trans-7,8-dihydrodiol and 3-OH-B[a]P-glucuronide were identified using the respective standards purchased from Toronto Chemicals Research (North York, Canada).

### Statistical analysis

All values were presented as means ± SEM (standard error of mean) from at least three independent experiments. Multiple comparisons among groups were performed using two-way analysis of variance (ANOVA) followed by a Bonferroni post-test, or one-way ANOVA followed by a Newman-Keuls post-test. To evaluate effects of HFD diet, one-tailed Student t-tests were performed. All statistical analyses were performed using GraphPad Prism5 software (GraphPad Software, San Diego, CA, USA). Differences were considered significant when P < 0.05. For cytotoxicity assay, 10% effective concentration (EC_10_) values were determined using GraphPad Prism software (GraphPad Software, LaJolla, CA).

### Data availability

The datasets generated during and/or analyzed during the current study are available from the corresponding author on reasonable request.

## Results

### Prior steatosis increases the long-term toxicity of B[a]P/ethanol co-exposure in the human hepatoma HepaRG cell line

**Figure 1:**
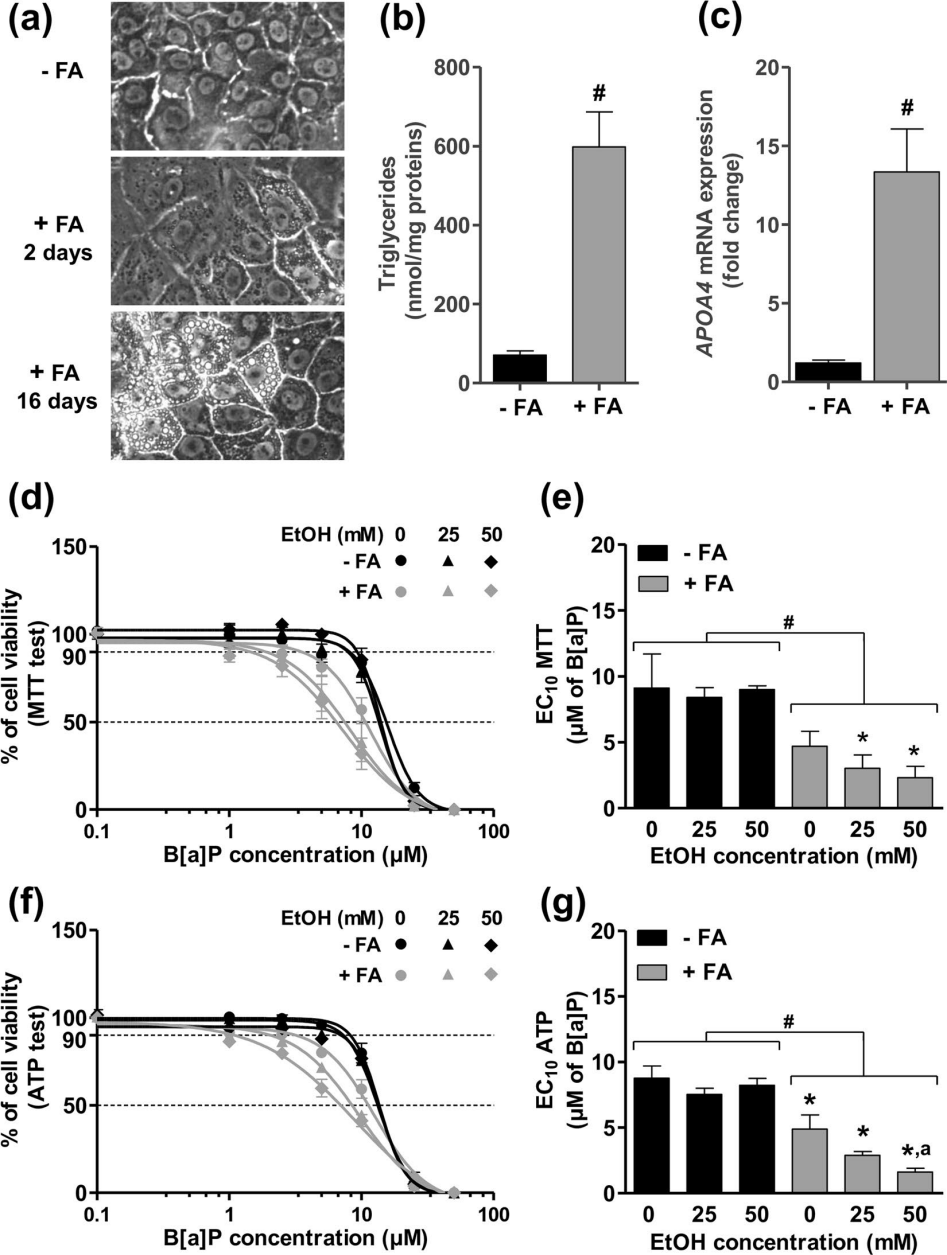
Toxicity of B[a]P in differentiated HepaRG cells is favored by steatosis and ethanol co-exposure. (**a**) Phase contrast microscopy of non-steatotic cells (−FA) and steatotic cells (+FA) after 2 and 16 days of incubation with a mixture of stearic acid and oleic acid. (**b**) Cellular triglyceride content in non-steatotic (−FA) and steatotic (+FA) cells after 16 days of FA overload. (**c**) mRNA levels of *APOA4* in non-steatotic (−FA) and steatotic (+FA) cells after 16 days of FA overload. (**d**) Cell viability determined by the MTT test in non-steatotic (−FA) and steatotic (+FA) cells exposed for 14 days to 0, 25 and 50 mM ethanol and a large range of B[a]P concentrations. (**e**) Corresponding B[a]P EC_10_ values in non-steatotic (-FA) and steatotic (+FA) cells exposed to 0, 25 and 50 mM ethanol. (**f,g**) Cell viability assessed by cellular ATP levels and corresponding B[a]P EC_10_ in non-steatotic (−FA) and steatotic (+FA) cells exposed for 14 days to 0, 25 and 50 mM ethanol and a large range of B[a]P concentrations. Results are means ± SEM for at least three independent cultures. (**b**,**c**) ^#^Significantly different from non-steatotic (−FA) cells. (**e**,**g**) ^#^Significantly different from non-steatotic cells; ^*^Significantly different from non-steatotic HepaRG cells treated by the same concentration of ethanol; ^a^Significantly different from steatotic HepaRG cells not treated by ethanol.

Recently, by using the metabolically competent human hepatoma HepaRG cell line, we have set up an *in vitro* human cell model of NAFLD for studying the toxicity of diverse drugs^33^. In order to evaluate the long-term effects of lifestyle-related toxicants, we therefore decided to use this model, especially as it is appropriate for such exposure times. First, this cell model was improved to further mimic the *in vivo* situation, notably by using a mixture of two FAs and by extending the duration of FA treatment to 16 days. Under these conditions, a lipid overload in cells was detected by microscopy as soon as 2 days of FA treatment, with a time-dependency as emphasized by an increase in the size and number of lipid droplets following 16 days of FA treatment (Fig. 1a). Lipid accumulation was further validated when measuring the triglyceride cell content, with a ∼6-fold increase in the presence of FAs as compared to control (Fig. 1b). A significant increase in *APOA4* mRNA expression was also observed in steatotic cells (Fig. 1c), as recently reported in the context of NAFLD^33,43^. Moreover, CYP2E1 activity was significantly enhanced by 50% in steatotic cells (data not shown), in keeping with several investigations performed in patients with NAFLD^44–46^.

The next set of experiments was carried out in order to determine the dose-response cytotoxicity curve of B[a]P, used alone or in combination with ethanol (25 or 50 mM), after a 14 day-treatment in steatotic and non-steatotic HepaRG cells. In non-steatotic cells, B[a]P toxicity, as evaluated by MTT test, remained unchanged by ethanol, whatever the concentration used (Fig. 1d). In steatotic cells, a marked leftward shift of the curve, that is a higher B[a]P cytotoxicity, was observed, with a further shift in presence of ethanol. This was clearly evidenced when calculating the EC_10_ for B[a]P cytotoxicity (Fig. 1e). Indeed, whereas no change in B[a]P EC_10_ was detected in control cells whatever the concentration of ethanol, a significant decrease in this EC_10_ was observed in steatotic cells (4.68 ± 0.95 *versus* 9.12 ± 2.24 µM in non-steatotic cells), which was further reduced by ethanol (3.12 ± 1.14 and 2.41 ± 0.84 µM for 25 and 50 mM ethanol, respectively). Similar results were obtained when measuring ATP concentration, with a significant difference already reached for 25 mM ethanol (3.07 ± 0.39 *versus* 4.83 ± 0.91 µM without ethanol; Fig. 1f,g). Altogether, these results clearly showed that a prior steatosis enhanced B[a]P cytotoxicity with an exacerbation of this effect in presence of ethanol. Based upon these results, subsequent investigations in HepaRG cells were carried out with 2.5 µM for B[a]P and 25 mM for ethanol. Notably, the high concentrations of B[a]P and ethanol used in differentiated HepaRG cells might be explained, at least in part, by high phase II and III XME activities^47–49^.

### Prior steatosis increases the toxicity of co-exposure to B[a]P/ethanol used at low concentrations in WIF-B9 cell line

In order to test if a sensitizing effect of steatosis could also be observed at concentrations of toxicants closer to human exposure, the hybrid WIF-B9 hepatic cell line was also used in this study. Indeed this cell line, which expresses both rat and human XMEs^27^, was recently shown by our group as reproducing the signaling cascade previously demonstrated in ethanol-treated primary rat hepatocytes^35,50,51^. Besides, it was found by McVicker and coworkers that co-exposing a parent cell line (WIF-B) to both ethanol and oleic acid markedly increased apoptosis when compared to ethanol alone^28^. Furthermore, it was shown that rat CYP1A1 and 1A2 were the most inducible CYPs (up to 100-fold with β-naphthoflavone) in WIF-B9 cells^27^. Finally, we previously found an increase in CYP2E1 activity upon ethanol treatment in control cells (unpublished data). Hence, all these data indicate that the WIF-B9 cell line is suitable to study ethanol and B[a]P metabolism and cytotoxicity.

**Figure 2:**
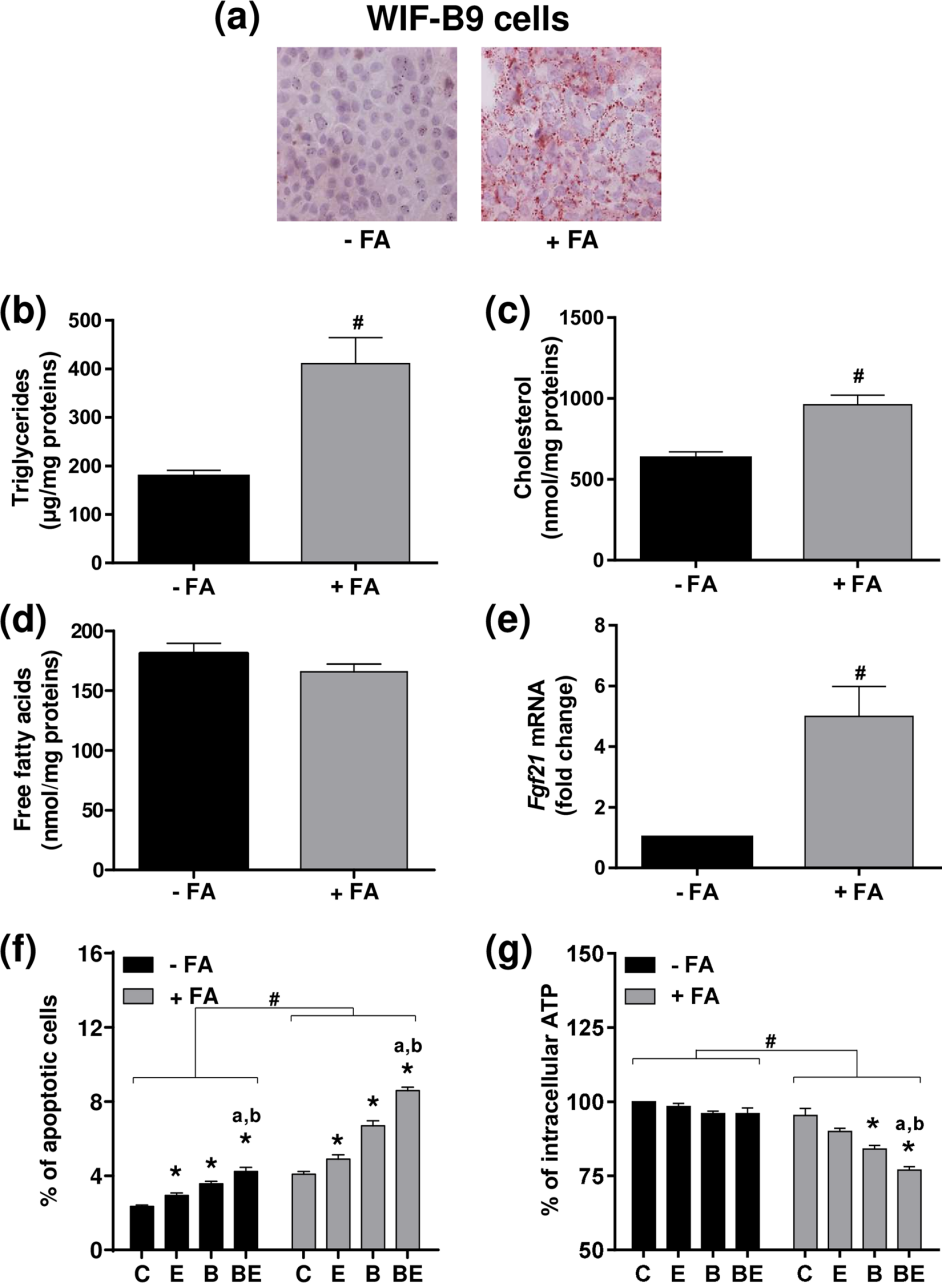
Toxicity of B[a]P in differentiated WIF-B9 cells is favored by steatosis and ethanol co-exposure. (**a**–**e**) Prior steatosis was induced by a 2 days incubation with palmitic acid and oleic acid (+FA conditions). (**a**) Fluorescence microscopy analysis of neutral lipid droplets after oil red O staining in non-steatotic (−FA) and steatotic (+FA) cells. Cellular triglyceride (**b**), cholesterol (**c**) and FFA (**d**) contents in non-steatotic (−FA) and steatotic (+FA). (**e**) mRNA levels of *Fgf21* in non-steatotic (−FA) and steatotic (+FA) cells. (**f**,**g**) Non-steatotic (−FA) and steatotic (+FA) cells were untreated (C) or treated with 10 nM B[a]P (B), 5 mM ethanol (E) or a combination of both toxicants (BE) for 5 days prior to evaluation of cytotoxicity by (**f**) counting apoptotic cells or (**g**) analyzing ATP content. Results are means ± SEM for at least three independent cultures. (**b**,**c**,**e**) ^#^Significantly different from non-steatotic cells. (**f**,**g**) ^#^Significantly different from non-steatotic cells; ^*^Significantly different from untreated non-steatotic or steatotic cells; ^a^Significantly different from non-steatotic or steatotic cells treated by ethanol only; ^b^Significantly different from non-steatotic or steatotic cells treated by B[a]P only.

A first set of experiments was thus performed in order to validate our FA overload protocol in WIF-B9 cells. Data from Fig. 2a–c showed that a 2 days exposure with a mixture of FAs increased the number of lipid droplets (a), and the triglyceride (b) and cholesterol (c) cellular contents. In contrast, no change was observed regarding the FFA content (Fig. 2d), in line with the very low toxicity detected under control conditions (Fig. 2f,g). Interestingly, the mRNA expression of fibroblast growth factor 21 (*Fgf21*), a known marker of NAFLD^52^, was markedly increased in steatotic WIF-B9 cells (Fig. 2e). All these data firmly validated our *in vitro* steatosis model.

Prior to testing the effects of B[a]P/ethanol co-exposure in steatotic WIF-B9 cells, experiments were performed in order to set the sub-toxic concentration of each toxicant used for subsequent investigations. Following a MTT test carried out after 5 days of treatment (Fig. S2), the selected concentrations were 10 nM and 5 mM, for B[a]P and ethanol, respectively, which is close to human exposure; indeed, up to 6.2 nM of B[a]P has been detected in sera of smoking women^53^; regarding alcohol, 5 mM [i.e. 0.23 g/l] is within the drinking guidelines for general populations published in 2017 by the International Alliance for Responsible Drinking [http://www.iard.org/policy-tables/drinking-guidelines-general-population/]). Cell toxicity was then evaluated by counting apoptotic cells (Fig. 2f) and measuring intracellular ATP content (Fig. 2g). Whereas the toxicity of chemicals alone or in co-exposure was low (albeit significant) after a 5 days exposure in non-steatotic WIF-B9 cells, the percentage of apoptotic cells markedly increased in steatotic cells (Fig. 2f). This was paralleled by a significant decrease in ATP content, especially in steatotic cells (Fig. 2g). It is worth noting that the toxicity of B[a]P/ethanol co-exposure was significantly higher than that of each toxicant alone. Altogether, these results showed that prior steatosis sensitized WIF-B9 hepatocytes to the toxicity of very low, sub-toxic concentrations of B[a]P and ethanol, with a stronger effect of co-exposure.

### Obese larvae exhibit high hepatotoxicity towards B[a]P/ethanol co-exposure

**Figure 3:**
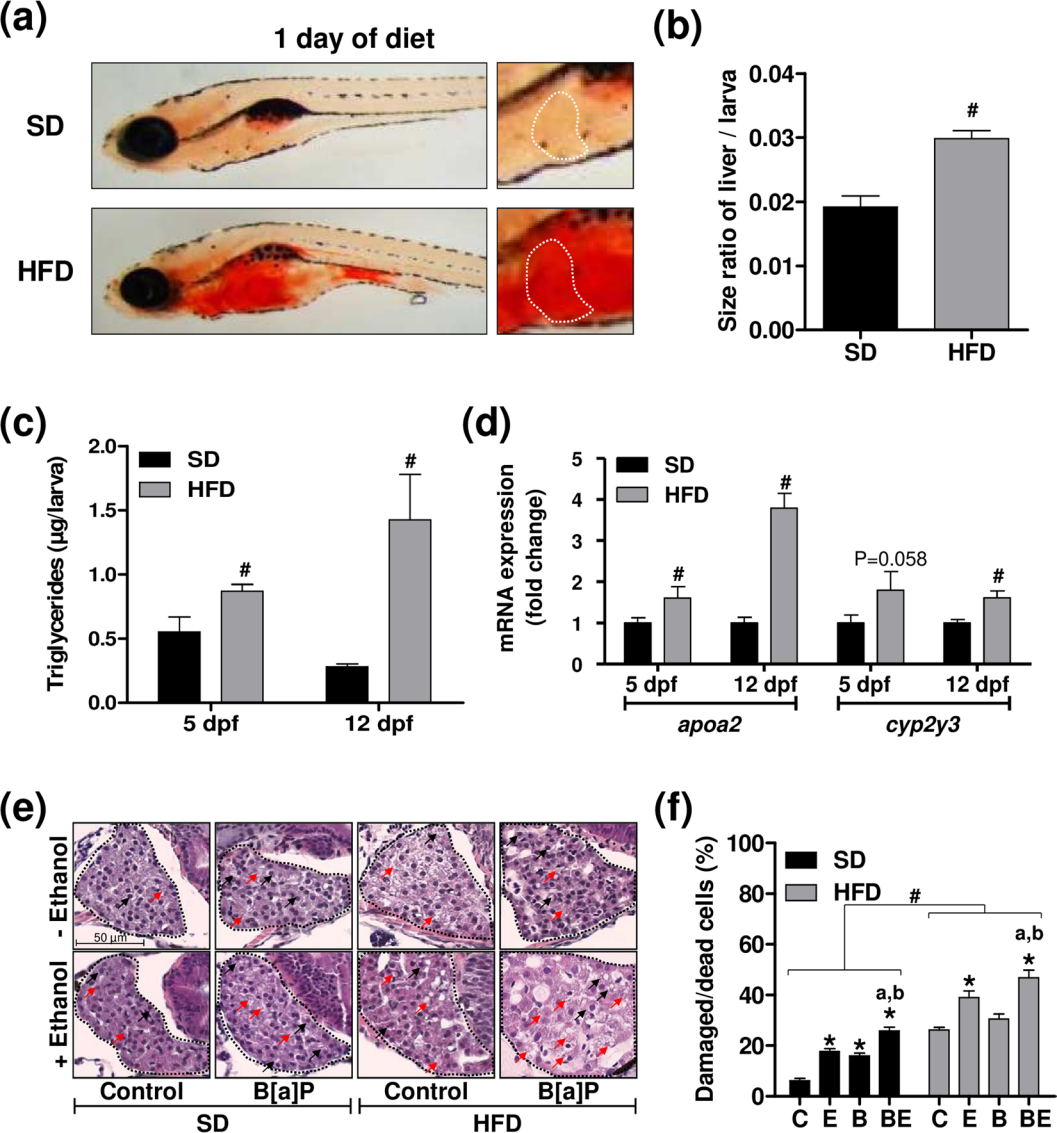
Induction of steatosis in zebrafish larvae under high-fat diet and exacerbation of liver damage severity upon co-exposure in larvae with steatosis. Zebrafish larvae were fed with a standard diet (SD) or a high-fat diet (HFD) from 4 dpf until 5 dpf (**a**–**d**) or until 12 dpf (**c**–**f**). Lipid accumulation (**a**) was analyzed after oil red O staining in HFD larvae as well as in SD larvae. White dotted line in the right-hand side panels outlines liver in the 2x-magnifications. (**b**) From images acquired in (**a**), the ratio of liver sizes to whole larva sizes was determined on 5 dpf zebrafish larvae. Images are representative of at least 3 larvae. Triglyceride content (**c**) as well as mRNA levels of *apoa2* and *cyp2y3* (**d**) were determined in SD and HFD larvae. In (**d**), data are expressed relative to mRNA level found in SD larvae, arbitrarily set at 1 unit for each time (5 and 12 dpf). (**e**,**f**) From 5 dpf, SD and HFD zebrafish were either left untreated (C), or treated with 25 nM B[a]P (B), 43 mM ethanol (E) or a combination of both toxicants (BE) for 7 days. (**e**) Liver damages were evaluated on zebrafish liver section after HES staining (magnification x400). Black dotted line outlines liver. Damaged/dead cells were indicated by red arrows for hepatocyte dropouts, and by black arrows for ballooned or vacuolated hepatocytes. Images are representative of at least 3 larvae. Values are the mean ± SEM of at least three independent experiments or larvae. (**f**) From images obtained in (**e**), histological count of damaged cells was realized. (**b**–**d**) ^#^Significantly different from SD larvae. (**f**) ^#^Significantly different from SD larvae; ^*^Significantly different from untreated SD or HFD larvae; ^a^Significantly different from larvae treated by ethanol only; ^b^Significantly different from SD or HFD larvae treated by B[a]P only.

In order to test whether steatosis could also enhance *in vivo* the hepatotoxicity of B[a]P/ethanol co-exposure, zebrafish larvae fed with a HFD (HFD larvae) were used as a suitable model for obesity-related NAFLD^54,55^. It is also noteworthy that ethanol can induce liver steatosis in zebrafish larvae^56,57^. We first showed that our feeding conditions did induce liver steatosis. Indeed, as shown in Fig. 3, a one-day HFD not only increased the oil red-O staining in liver (a) but also the size of liver relatively to whole body (b), when compared to standard diet (SD). Regarding a potential interference with adipose tissue on the oil red-O staining at 5 dpf, it could be easily discarded as adipose tissue in larvae is known to appear only from 8 dpf ^58,59^. The triglyceride content of whole larvae was also found to increase upon HFD from one day of HFD (5dpf), with a marked effect observed after 8 days of diet (12 dpf) (Fig. 3c). The mRNA levels of *apoa2* and *cyp2y3* (homologous to the human *CYP2E1* gene), two genes whose expression is modulated during NAFLD^46,60^, were enhanced in whole HFD larvae (Fig. 3d).

For subsequent investigations, 25 nM B[a]P and 43 mM ethanol (corresponding to 10 mM ethanol inside larvae; data not shown) were chosen as these concentrations induced a very low mortality within the SD larvae population following 7 days of treatment (Fig. S3). In order to evaluate hepatotoxicity, a histological analysis was performed. HFD markedly increased liver alterations upon B[a]P/ethanol co-exposure when compared to SD, with an increase in the number of damaged/dead cells (Fig. 3e). This was clearly visualized on the histogram plotting the number of damaged/dead cells counted under the different experimental conditions (Fig. 3f). Importantly, toxicity of B[a]P/ethanol co-exposure was significantly higher compared to each chemical alone. Therefore, the *in vivo* steatosis also sensitizes liver to B[a]P/ethanol-related toxicity.

### B[a]P/ethanol co-exposure triggers inflammation in NAFLD both *in vitro* and *in vivo*

**Figure 4:**
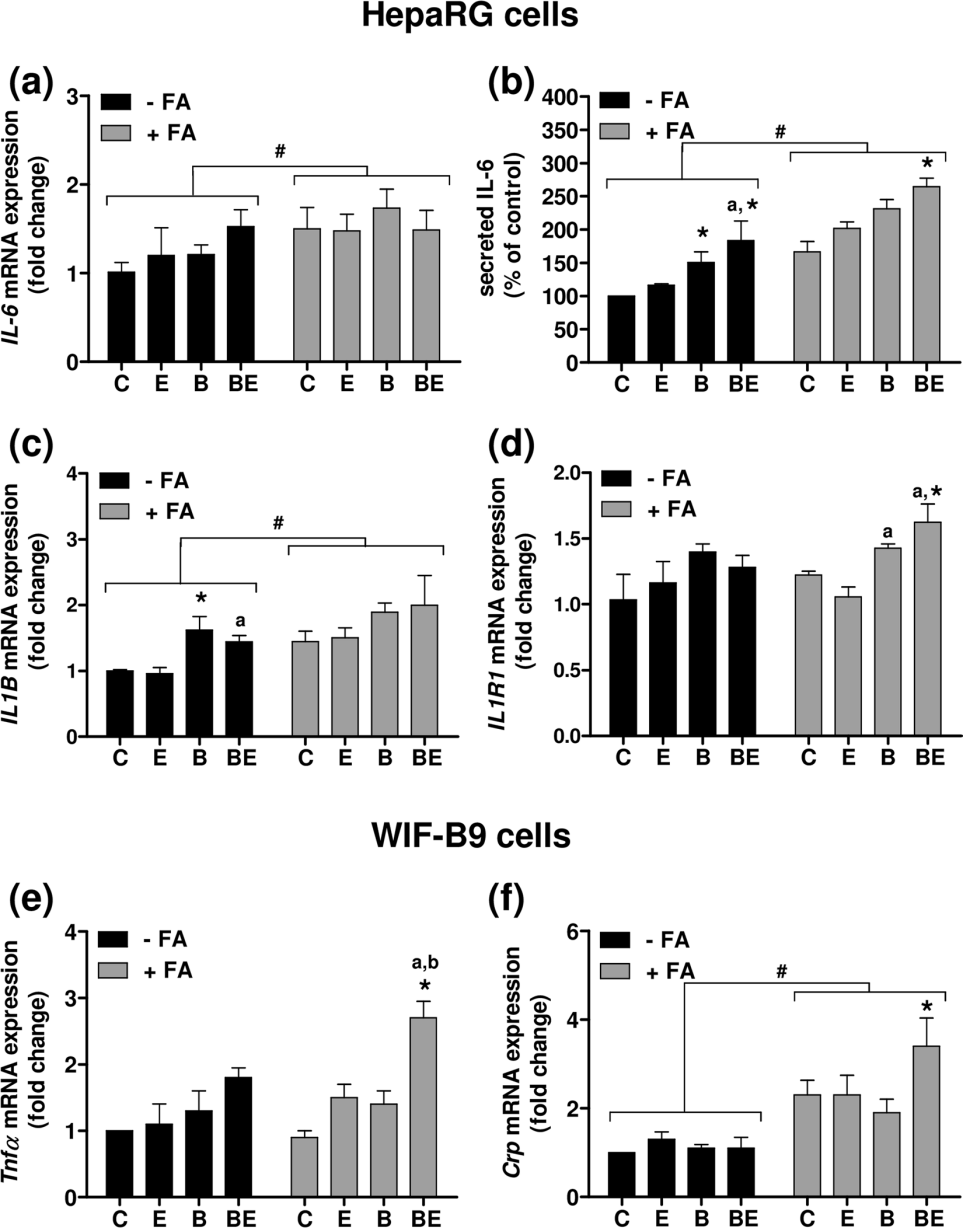
B[a]P/ethanol co-exposure favors a pro-inflammatory state in steatotic HepaRG and WIF-B9 cells. (**a**–**d**) Non-steatotic (−FA) and steatotic (+FA) HepaRG cells were untreated (C) or treated with 2.5 µM B[a]P (B), 25 mM ethanol (E) or a combination of both toxicants (BE). (**e**,**f**) Non-steatotic (−FA) and steatotic (+FA) WIF-B9 cells were untreated (C) or treated with 10 nM B[a]P (B), 5 mM ethanol (E) or a combination of both toxicants (BE). (**a**,**c**,**d**) mRNA expression of *IL-6*, *IL1B* and *IL1R1*. (**b**) Secreted IL6 levels in the culture medium. (**e**,**f**) mRNA expression of *Tnfα* and *Crp*. Results are means ± SEM for at least three independent cultures. ^#^Significantly different from non-steatotic cells; ^*^Significantly different from untreated non-steatotic or steatotic cells; ^a^Significantly different from non-steatotic or steatotic cells treated by ethanol only; ^b^Significantly different from non-steatotic or steatotic cells treated by B[a]P only.

The next set of experiments was performed in order to test whether the increased toxicity of B[a]P/ethanol co-exposure was paralleled by the onset of an inflammatory state. First, the expression of several pro-inflammatory cytokines was analyzed in both *in vitro* cell models. In HepaRG cells treated or not with B[a]P (2.5 µM) and/or ethanol (25 mM) for 14 days, a significant increase in interleukin 6 (*IL6)* and interleukin 1β (*IL1β)* mRNA expression was observed in steatotic cells as compared to non-steatotic cells (Fig. 4a,c). However, no significant effect of toxicants, used alone or in co-exposure, was detected in steatotic cells. Nevertheless, secreted IL6 levels were enhanced, especially upon B[a]P/ethanol co-exposure, with a stronger effect detected in steatotic HepaRG cells (Fig. 4b). Regarding the IL1β pathway, a significant increase in mRNA expression of the IL1β receptor *IL1R1* was also detected especially upon toxicant co-exposure in steatotic cells (Fig. 4d).

In WIF-B9 cells treated or not by B[a]P (10 nM) and/or ethanol (5 mM) for 5 days, a significant increase in tumor necrosis factor α *(Tnfα)* mRNA expression was found (by ∼2.8-fold) upon toxicant co-exposure in steatotic cells (Fig. 4e). Such an onset of inflammation in steatotic WIF-B9 cells was confirmed by analyzing the mRNA expression of *Crp* (Fig. 4f), a well-known marker of inflammation^61^. Indeed, *Crp* mRNA expression was higher not only in presence of steatosis, but was further increased when steatotic cells were co-treated with B[a]P/ethanol.

**Figure 5:**
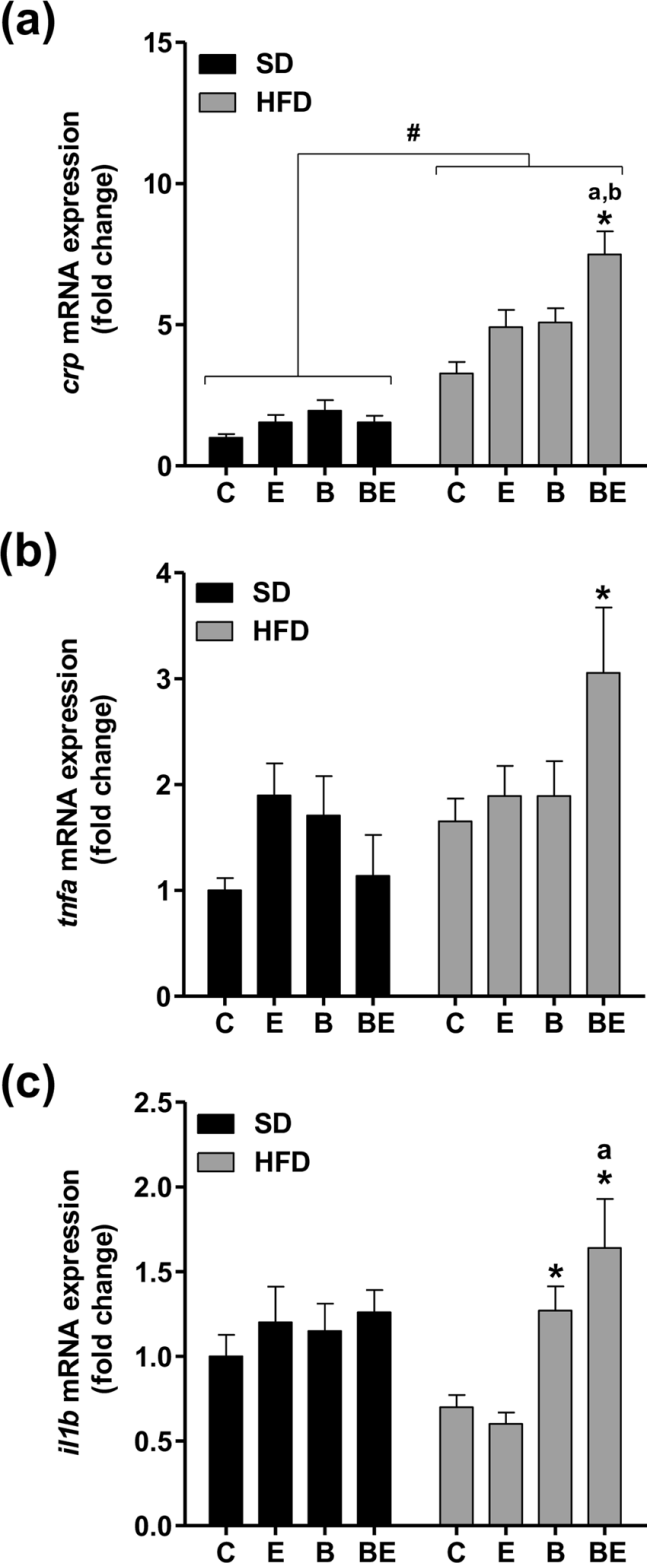
B[a]P/ethanol co-exposure favors a pro-inflammatory state in in HFD zebrafish larvae. Zebrafish larvae were fed with a standard diet (SD) or a high fat diet (HFD) and were either left untreated (C), or treated with 25 nM B[a]P (B), 43 mM ethanol (E) or a combination of both toxicants (BE) for 7 days. (**a**–**c**) mRNA expression of *crp, tnfa* and *il1b*, respectively. Data are expressed relative to mRNA level found in control SD larvae, arbitrarily set at 1 unit. Values are the mean ± SEM of at least twelve independent experiments. ^#^Significantly different from SD larvae; ^*^Significantly different from untreated SD or HFD larvae; ^a^Significantly different from larvae treated by ethanol only; ^b^Significantly different from SD or HFD larvae treated by B[a]P only.

Regarding the *in vivo* model, the co-exposure of zebrafish larvae to B[a]P (25 nM)/ethanol (43 mM) for 7 days resulted in a significant increase in *crp, tnfa* and *il1b* mRNA expression in whole animals but only under HFD conditions (Fig. 5a–c), thus corroborating the effects observed *in vitro*. Note that *crp* mRNA expression was significantly higher in HFD larvae compared to SD larvae in the absence of any treatment (Fig. 5a). In line with the results obtained from whole larvae, hepatic *crp* mRNA expression in HFD larvae was higher with B[a]P/ethanol co-exposure compared to each toxicant alone (Fig. S4).

### Effects of steatosis and ethanol co-exposure on phase I and II XMEs in HepaRG cells

**Figure 6:**
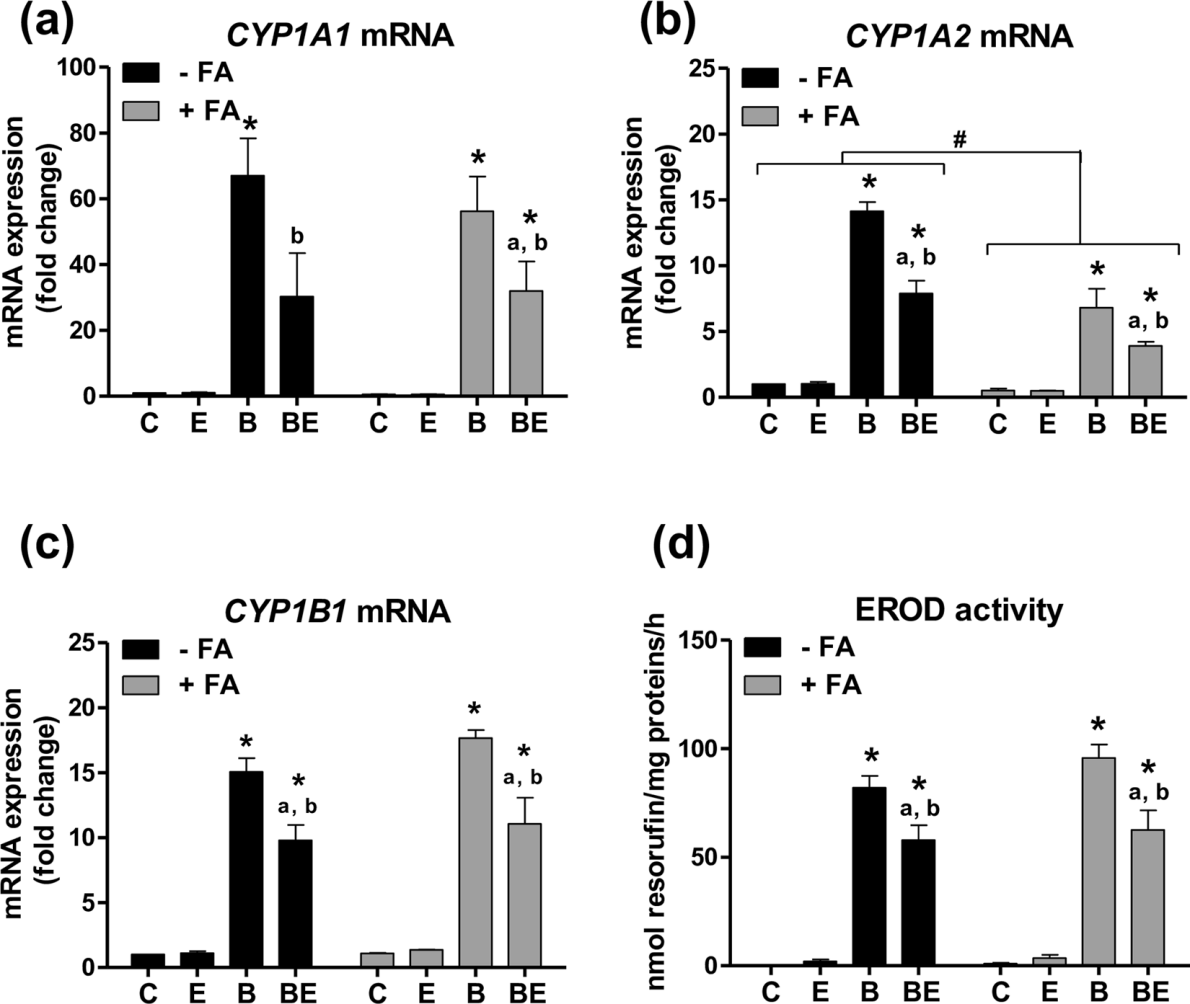
mRNA expression of *CYP1A1, CYP1A2* and *CYP1B1* and EROD activity are disturbed in non-steatotic and steatotic HepaRG cells treated with B[a]P and ethanol. Non-steatotic (-FA) and steatotic (+FA) HepaRG cells were untreated (C) or treated with 2.5 µM B[a]P (B), 25 mM ethanol (E) or a combination of both toxicants (BE). (**a**–**c**) mRNA expression of *CYP1A1*, *CYP1A2* and *CYP1B1*. (**d**) EROD activity. Results are means ± SEM for at least three independent cultures. ^#^Significantly different from non-steatotic cells; ^*^Significantly different from untreated non-steatotic or steatotic cells; ^a^Significantly different from non-steatotic or steatotic cells treated by ethanol only; ^b^Significantly different from non-steatotic or steatotic cells treated by B[a]P only.

In order to get insight into the possible mechanisms involved in the sensitizing effects of steatosis and ethanol co-exposure towards B[a]P toxicity, we performed a series of investigations in HepaRG cells to determine whether lipid overload and ethanol could impair the expression of the main XMEs involved in B[a]P metabolism, especially CYP1A1, 1A2 and 1B1. As expected, the mRNA expression of these CYPs was markedly enhanced after 14 days of treatment with 2.5 µM B[a]P in steatotic and non-steatotic HepaRG cells, with the strongest effect observed for *CYP1A1* (Fig. 6a–c). Of note, increased *CYP1A2* mRNA expression was lesser in the presence of steatosis (Fig. 6b). Moreover, mRNA expression *of CYP1A1, 1A2* and *1B1* was significantly decreased by 25 mM ethanol co-exposure both in steatotic and non-steatotic cells (Fig. 6a–c). Next, EROD activity was assessed in order to evaluate the overall activity of these CYPs. EROD activity was markedly increased by B[a]P but no difference was observed between steatotic and non-steatotic HepaRG cells (Fig. 6d). However, ethanol co-exposure resulted in a lesser increase of EROD activity (with similar effects when comparing steatotic and non-steatotic cells), thus reflecting the mRNA expression profile of *CYP1A1* and *1B1*. Therefore, it appears that the activation of the CYP1 pathway by B[a]P alone or with ethanol was not affected by prior steatosis in HepaRG cells.

We also took advantage of a whole-genome transcriptome analysis (GSE102536 – see supplementary Methods for protocol) to determine whether steatosis and ethanol altered the expression of other XMEs involved or not in B[a]P biotransformation (Table S2). Notably, this analysis confirmed our results regarding the mRNA expression of *CYP1A1, 1A2* and *1B1*, especially the lower expression of *CYP1A2* in steatotic HepaRG cells treated or not with B[a]P and ethanol. Furthermore, the transcriptome analysis clearly showed that lipid overload repressed the expression of other phase I XMEs involved in B[a]P metabolism such as *CYP3A4, CYP2C19*, aldo-keto reductases (AKRs) and epoxide hydrolases (EPHXs)^19,62^. Steatosis also induced a downregulation of several phase II XMEs involved in B[a]P detoxification including glutathione-S-transferases (GSTs), sulfotransferases (SULTs) and UDP-glucuronosyl transferases (UGTs), as well as a downregulation of XMEs involved in ethanol metabolism such as alcohol dehydrogenases (ADHs) and aldehyde dehydrogenases (ALDHs). The mRNA expression of *CYP2E1* was however increased in steatotic HepaRG cells in the absence of any treatment. It was also noteworthy that, among the 12 experimental conditions tested, the lowest mRNA expression of phase I and phase II XMEs was mostly observed in steatotic HepaRG cells treated with 2.5 µM B[a]P. Moreover, the lowest expression of several phase II XMEs such as GSTM2P1, GSTA7P, SULT1B1, SULT1C2, UGT2B7 and UGT2A3 was observed in steatotic HepaRG cells co-exposed to 2.5 µM B[a]P and 25 mM ethanol (Table S2).

### Effects of steatosis and ethanol co-exposure on the amount of B[a]P metabolites produced in HepaRG cells

Based upon the above results, it appeared that the whole B[a]P metabolism might be altered in steatotic HepaRG cells exposed to ethanol. Hence, at the end of the 14-day exposure and after a 15-min washout, we assessed the formation of B[a]P metabolites after an acute incubation of cells with 25 µM B[a]P. Importantly, this analysis was performed not only in steatotic and non-steatotic HepaRG cells treated for 14 days with 2.5 µM B[a]P with or without ethanol but also in cells not previously exposed to this toxicant. Thus, the ability of HepaRG cells to metabolize B[a]P was determined even in cells that have not been chronically exposed to B[a]P.

**Figure 7:**
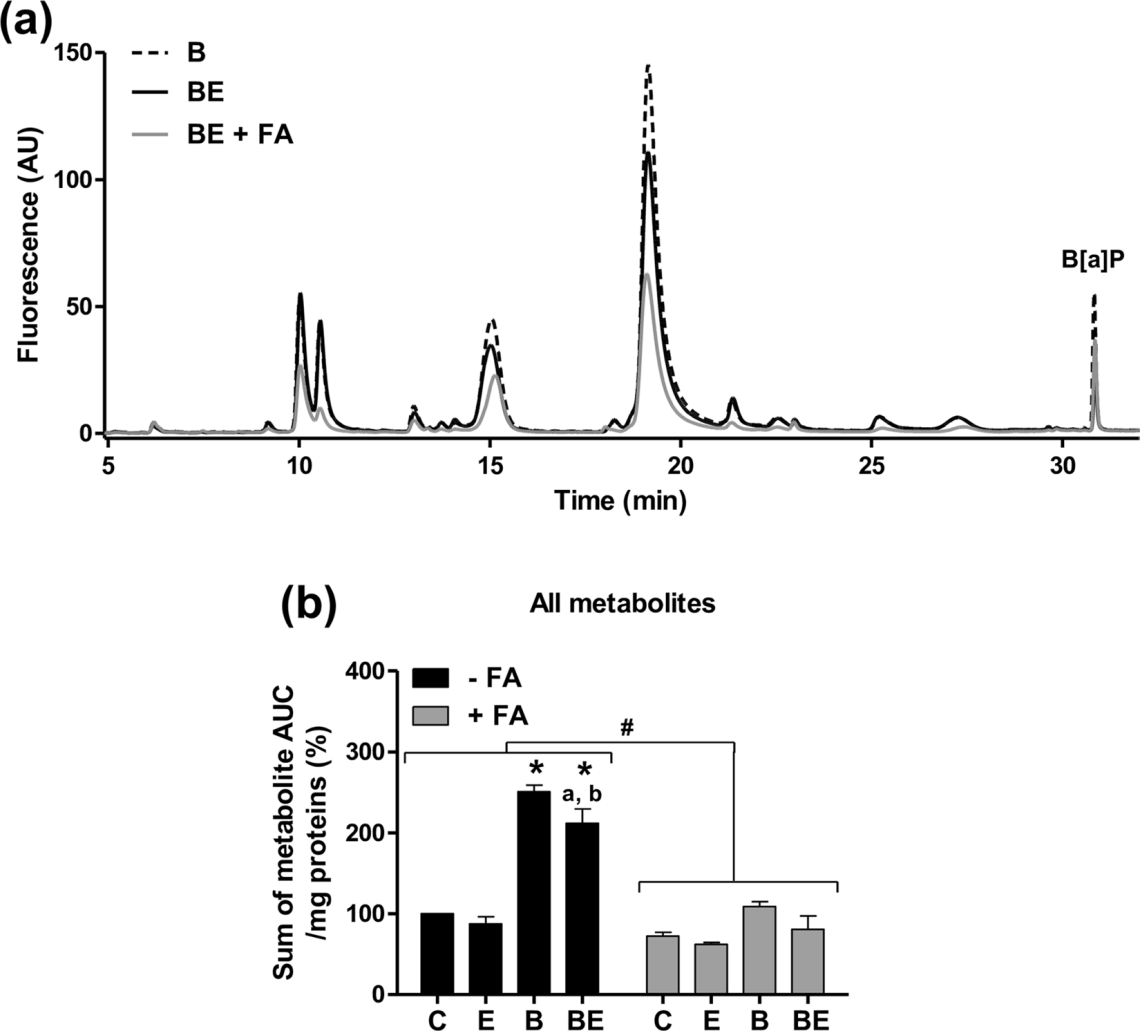
B[a]P metabolism is disturbed in HepaRG cells by steatosis and ethanol co-exposure. Non-steatotic (−FA) and steatotic (+FA) HepaRG cells were untreated (C) or treated with 2.5 µM B[a]P (B), 25 mM ethanol (E) or a combination of both toxicants (BE). At the end of the 14-day toxicant exposure and after a 15-minute washout, B[a]P metabolites were analyzed in the culture media after an acute incubation of 25 µM B[a]P. (**a**) Examples of three representative HPLC chromatograms corresponding to non-steatotic cells exposed to B[a]P (B), non-steatotic cells co-exposed to B[a]P and ethanol (BE), and steatotic cells co-exposed to B[a]P and ethanol (BE + FA). (**b**) Amount of all detected B[a]P metabolites determined as the sum of their AUCs (areas under the curve) and normalized to the total cellular protein content. Results are means ± SEM for at least three independent cultures. ^#^Significantly different from non-steatotic cells; ^*^Significantly different from untreated non-steatotic cells; ^a^Significantly different from non-steatotic cells treated by ethanol only; ^b^Significantly different from non-steatotic cells treated by B[a]P only.

Examples of three representative HPLC chromatograms are shown in Fig. 7a, corresponding to non-steatotic cells exposed to B[a]P, non-steatotic cells co-exposed to B[a]P/ethanol, and steatotic cells co-exposed to B[a]P/ethanol. Several peaks could be identified on these HPLC chromatograms, with a clear reduction of the overall amount of B[a]P metabolites by ethanol co-exposure and a further decrease in the presence of steatosis (Fig. 7a). Notably, steatosis-induced reduction of all detected B[a]P metabolites was observed under the different experimental conditions when the amount of B[a]P metabolites was assessed using the areas under the curve (AUC) (Fig. 7b).

**Figure 8:**
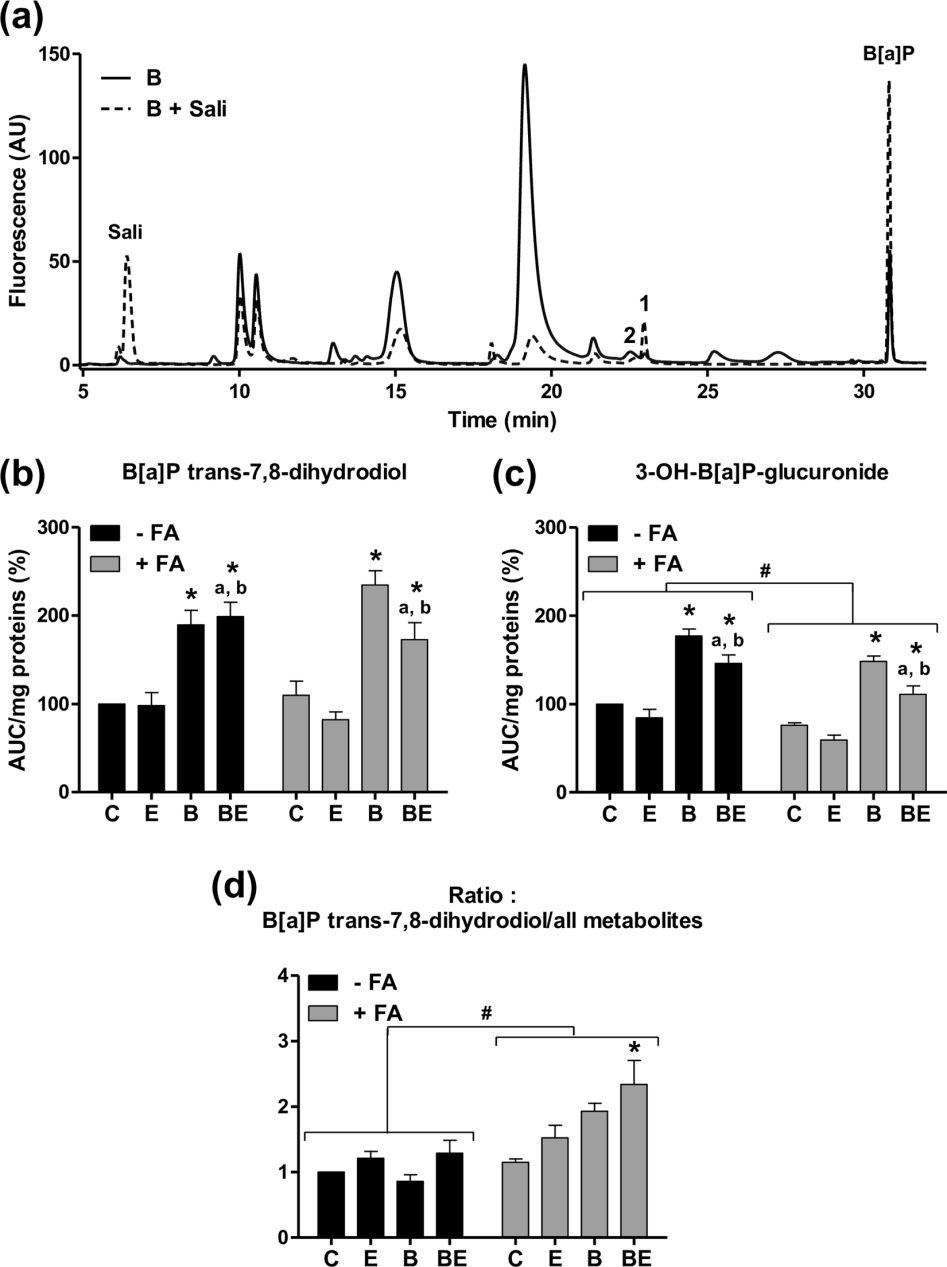
B[a]P metabolism by phase I and II XMEs is disturbed in HepaRG cells by steatosis and ethanol co-exposure. Non-steatotic (−FA) and steatotic (+FA) HepaRG cells were untreated (C) or treated with 2.5 µM B[a]P (B), 25 mM ethanol (E) or a combination of both toxicants (BE). At the end of the 14-day toxicant exposure and after a 15-minute washout, B[a]P metabolites were analyzed in the culture media after an acute incubation of 25 µM B[a]P with or without 5 mM salicylamide, a strong inhibitor of phase II XMEs. (**a**) Examples of two representative HPLC chromatograms corresponding to non-steatotic cells treated for 14 days with B[a]P, and then acutely exposed to B[a]P (B) or B[a]P with salicylamide (B + Sali). (**b**) Amount of B[a]P trans-7,8-dihydrodiol (peak 1 on panel ***a***) assessed by its AUC and normalized to the total cellular protein content. (**c**) Amount of 3-OH-B[a]P-glucuronide (peak 2 on panel ***a***) assessed by its AUC and normalized to the total cellular protein content. (**d**) Ratio of the amount of B[a]P trans-7,8-dihydrodiol to the amount of all detected metabolites. Results are means ± SEM for at least three independent cultures. ^#^Significantly different from non-steatotic cells; ^*^Significantly different from untreated non-steatotic or steatotic cells; ^a^Significantly different from non-steatotic or steatotic cells treated by ethanol only; ^b^Significantly different from non-steatotic or steatotic cells treated by B[a]P only.

In order to get further information regarding the nature of the detected B[a]P metabolites, we next performed investigations in HepaRG cells acutely exposed to 25 µM B[a]P and 5 mM salicylamide, a known inhibitor of phase II enzymes. Interestingly, most of the peaks were reduced by salicylamide (Fig. 8a), thus indicating that they corresponded to metabolites produced by phase II XMEs. We also identified two important B[a]P metabolites, namely 3-OH-B[a]P-glucuronide and B[a]P trans-7,8-dihydrodiol (Fig. S5), using the corresponding standards (respectively metabolites 2 and 1 on the chromatograms in Fig. 8A). Notably, salicylamide treatment reduced the peak corresponding to 3-OH-B[a]P-glucuronide and concomitantly increased the peak corresponding to B[a]P trans-7,8-dihydrodiol (Fig. S6), a precursor of several toxic B[a]P metabolites (Fig. S5).

The amounts of B[a]P trans-7,8-dihydrodiol and 3-OH-B[a]P-glucuronide were next assessed. Whereas no significant difference in the amount of B[a]P trans-7,8-dihydrodiol could be observed between steatotic and non-steatotic HepaRG cells (Fig. 8b), that of 3-OH-B[a]P-glucuronide was significantly reduced in the presence of steatosis (Fig. 8c). It was worth noting that a significant decrease in the amount of both metabolites was detected in steatotic HepaRG cells co-exposed to B[a]P/ethanol as compared to steatotic cells treated with B[a]P alone (Fig. 8b,c). Interestingly, the ratio of B[a]P trans-7,8-dihydrodiol level to the amount of all metabolites was found markedly enhanced in steatotic HepaRG cells co-exposed to B[a]P/ethanol (Fig. 8d). Altogether, these results suggested that steatosis and ethanol co-exposure could induce a shift in B[a]P metabolism with an impairment of its detoxification.

## Discussion

Hepatic steatosis and steatohepatitis have been related to diverse etiologic factors, the most frequent being alcohol (ALD), obesity (NAFLD), and environmental toxicants (TASH)^9,13^. However, to our knowledge, no study has been performed so far with the aim of analyzing how these three different factors might interplay with respect to the progression of liver diseases. In this context, we decided to test the impact of the co-exposure to both the environmental carcinogen B[a]P and the lifestyle-related hepatotoxicant ethanol, on different models of hepatic steatosis induced by either FA overload (*in vitro*) or HFD (*in vivo*). The present study shows for the first time that the presence of a prior steatosis significantly enhanced the toxicity of B[a]P/ethanol co-exposure, and that such a co-exposure might favor the appearance of a steatohepatitis-like state, even at concentrations determined as sub-toxic under FA-free conditions.

Using two different *in vitro* models of steatosis, a significant increase in cell death (notably associated with a decrease in intracellular ATP content) was detected upon co-exposure to both toxicants. This cytotoxicity was associated with an increase in the mRNA expression of some cytokines (*IL1β* and its receptor *IL1R1*, in HepaRG cells; *Tnfα* in WIF-B9 cells), as well as in *Crp* mRNA expression in these latter cells. Secreted IL6 was also detected in HepaRG cells. This therefore might indicate the onset of inflammation. The differences observed with regard to the type of altered cytokines between HepaRG and WIF-B9 cells might stem from either the concentrations of toxicants (B[a]P/ethanol: 2.5 µM/25 mM for HepaRG cells *versus* 10 nM/5 mM for WIF-B9 cells), the time of co-exposure (14 days for HepaRG cells *versus* 5 days for WIF-B9 cells), or interspecies features (human for HepaRG cells *versus* hybrid human/rat for WIF-B9 cells).

Notably, an increase in cell death as well as in the expression of several markers of inflammation (*crp, tnfa, il1b*), was also found in HFD zebrafish larvae co-exposed to B[a]P and ethanol. Zebrafish larvae possess a functional immune system (as evidenced by neutrophil recruitment^63^), thus showing that the pathological progression of steatosis observed in our two *in vitro* models could also be seen in an *in vivo* model of NAFLD which is closer to the clinical situation. These observations not only emphasize the utility of the two *in vitro* models of steatosis presently developed, as already reported^28,33^, but also further reinforce the attractiveness of the zebrafish larvae as a suitable model to study xenobiotic-related liver diseases^35^. Moreover, the fact that the steatohepatitis-like state was observed with different FA mixtures (*in vitro*) and different lipids (*in vivo*) strengthens the robustness of our experimental results and their possible extrapolation to NAFLD patients who are likely to eat different types of high-fat diets.

It was previously shown that alcohol intoxication in the context of obesity was able to aggravate NAFLD^14,15^. Similarly, recent data demonstrated that endocrine disruptors, such as bisphenol A or PCB153, could also worsen NAFLD when promoted by high fat diet^9^. In the present study, at the concentrations tested, the effects of ethanol or B[a]P alone in steatotic cells were quite minor, if any, especially on inflammation markers. From our data, it therefore clearly appears that this is the combination of all three risk factors (obesity, alcohol consumption, exposure to environmental toxicants) that can enhance the risk of fatty liver disease progression. This might give a clue as to why there has been a large increase in the incidence of fatty liver diseases throughout the past two decades, accompanied by an increased risk of HCC among patients with NAFLD^5^.

We previously demonstrated in primary rat hepatocytes that the cooperative interaction between B[a]P and ethanol on cell death involved both B[a]P and ethanol metabolism^25^. Besides, a few studies indicated an impact of liver steatosis on xenobiotic metabolism, with possible consequences on drug biotransformation^64–69^, and toxicokinetics of environmental contaminants^70^. We therefore decided to focus on xenobiotic metabolism in HepaRG cells, especially that related to B[a]P. As expected from previous works^64–67^, steatosis *per se* down-regulated the expression of several phase I and II XMEs of HepaRG cells, with some exceptions such as *CYP2E1, ALDH1A3* and *GSTM2P1* whose expression was increased (Table S2). Such *CYP2E1* induction has already been reported in clinical and experimental NAFLD^33,44,46^.

Regarding more specifically B[a]P metabolism, it is worth noting that neither *CYP1A1* nor *CYP1B1* mRNA expressions were affected by steatosis alone, in contrast to *CYP1A2* whose expression was reduced. Despite a marked induction of these CYPs by B[a]P, ethanol however decreased it in both steatotic and non-steatotic cells. One might propose that such an impact of ethanol would be related to its inducing effect on *CYP2E1* in HepaRG cells. Indeed, it has been previously shown that CYP2E1 overexpression repressed the activity of the *CYP1A1* gene promoter and *vice versa*, *via* a cross-regulation involving reactive oxygen species production between those two enzymes^71,72^. However, when looking at the present transcriptomics data, it appears that like *CYP1A1, CYP2E1* mRNA expression was also down-regulated in cells exposed to B[a]P/ethanol mixture (Table S2), and so was the activity of both CYP1A1 (Fig. 6d) and CYP2E1 (data not shown), especially in steatotic cells. Based upon the fact that the mRNA expression of several phase I and phase II XMEs was affected in steatotic cells exposed to both B[a]P and ethanol (Table S2), pathophysiological parameters such as oxidative stress^72,73^, inflammation^74,75^, or lipid accumulation^68,69^, might be involved in these effects, e.g. by controlling the activity or expression of key nuclear receptors. With regard to an effect of inflammation, it has been previously reported that CYP1A2 expression is decreased in the presence of pro-inflammatory cytokines such as TNFα and IL1β, likely through an effect on the aryl hydrocarbon receptor (AhR)^76^. Such a mechanism might be involved in the decrease in *CYP1A2* mRNA expression presently observed in steatotic HepaRG cells since the IL1β pathway was upregulated.

The fact that xenobiotic metabolism was altered by steatosis and toxicant co-exposure led us to analyze the B[a]P metabolites produced under our different conditions. From the present results, it was clear that far less metabolites were produced by steatotic HepaRG cells following the 14 days treatment with both B[a]P (2.5 µM) and ethanol (25 mM) as compared to non-steatotic cells (Fig. 7b). Such a decrease in the overall amount of metabolites might result from the reduced expression of the enzymes involved in B[a]P biotransformation, as discussed above. As EROD activity did not seem to be affected by steatosis whatever the test conditions, one might then suppose that enzymes other than CYP1 would be targeted. In line with this, our transcriptomic analysis evidenced in steatotic cells a reduced expression of several enzymes involved in B[a]P metabolism including CYP3A4 and 2C19 as well as AKRs, EPHXs, GSTs and UGTs^77,78^. Moreover, a significant reduction in the amount of 3-OH-B[a]P-glucuronide was observed in steatotic cells (Fig. 8c), thus indicating that B[a]P detoxification via the UGT pathway would be impaired. Interestingly, previous works showed a decrease in the activity of phase II XMEs as NAFLD progresses from steatosis to steatohepatitis^79^.

It is noteworthy that B[a]P trans-7,8-dihydrodiol is the precursor of (±)-anti-B[a]P-diol-epoxide (BPDE) (Figure S4), the major carcinogenic intermediate of B[a]P^80^. Our results showing an increase in the ratio of B[a]P trans-7,8-dihydrodiol/all metabolites in steatotic cells co-exposed to B[a]P/ethanol (Fig. 8d) thus suggested an impairment of B[a]P detoxification. As a consequence, one might then expect higher formation of BPDE-DNA adducts and other DNA damages, eventually leading to an increased cell toxicity and higher risk of carcinogenesis. In addition to DNA damages, it would also be interesting to analyze the DNA repair systems. Indeed, a recent study dealing with the impact of acidic pH on B[a]P metabolism demonstrated a delayed B[a]P metabolism associated with a decreased DNA repair activity, ultimately leading to higher DNA damage and DNA adduct formation^81^.

In conclusion, we presently report for the first time that a co-exposure to B[a]P/ethanol favors *in vitro* and *in vivo* the progression of fatty liver to a more severe stage characterized by cytotoxicity and a pro-inflammatory state. This progression seems to be promoted by a profound effect of steatosis and ethanol on the expression on phase I and II XMEs leading to a change in the balance between B[a]P bioactivation and detoxification. Based upon the fact that NAFLD is a growing public health burden, associated with a significant economic impact^1,7^, elucidation of the mechanisms whereby B[a]P and ethanol co-exposure aggravate NAFLD will have to be thoroughly tackled in the near future.

## Electronic supplementary material

Supplementary information
