# Supplementary material for: Co-exposure to benzo[a]pyrene and ethanol induces a pathological progression of liver steatosis in vitro and in vivo
Source: Sci Rep. 2018 Apr 13;8:5963. doi: 10.1038/s41598-018-24403-1 (PMC5899096; doi:10.1038/s41598-018-24403-1)
Supplement: Supplementary file 1 — Supplementary information [file 41598_2018_24403_MOESM1_ESM.pdf]

SUPPLEMENTARY INFORMATION

Co-exposure to benzo[a]pyrene and ethanol induces a pathological progression of liver steatosis *in vitro* and *in vivo*

Simon Bucher[1]*, Arnaud Tête[2]*, Normand Podechard[2]*, Marie Liamin[2], Dounia Le Guillou[1], Martine Chevanne[2], Cédric Coulouarn[1], Muhammad Imran[2], Isabelle Gallais[2], Morgane Fernier[2], Quentin Hamdaoui[1], Marie-Anne Robin[1], Odile Sergent[2#], Bernard Fromenty[1#], Dominique Lagadic-Gossmann[2#$]

SUPPLEMENTARY METHODS

Chemicals

B[a]P, dimethylsulfoxide (DMSO), Hoechst 33342, Sytox Green, Oil Red O, insulin, (3-(4,5-dimethylthiazol-2-yl)-2,5-diphenyltetrazolium bromide) (MTT), oleic acid, stearic acid, palmitic acid, salicylamide, and triethylamine were purchased from Sigma Aldrich (Saint-Quentin-Fallavier, France). Fetal Bovine Serum (FBS) was purchased from Lonza (Levallois-Perret, France), and Fetal Calf Serum (FCS) from Eurobio (Courtaboeuf, France). Ethanol was purchased from Prolabo (Paris, France). For HepaRG cell culture, William's E medium, glutamine, penicillin and streptomycin were obtained from Thermo Fisher Scientific (Cergy Pontoise, France). Hydrocortisone hemisuccinate was purchased from Serb Laboratories (Paris, France). For WIF-B9 cell culture, F-12 Ham medium with Coon's modification, penicillin, streptomycin and amphotericin B were purchased from Sigma Aldrich, and glutamine and HAT (hypoxanthine, aminopterin, thymidine) were obtained from Gibco (Cergy Pontoise, France). B[a]P trans-7,8-dihydrodiol and 3-hydroxy-B[a]P-glucuronide (3-OH-B[a]P) were obtained from Toronto Chemicals Research (North York, Canada).

Transcriptomic analysis in HepaRG cells

Genome-wide expression profiling was performed using the low-input QuickAmp labeling kit and SurePrint G3 human GE v3 8x60K microarrays purchased from Agilent Technologies (Santa Clara, CA), as previously described (Coulouarn et al. 2012). Total RNA, in presence of an external spike-in RNA mixture, were amplified and labeled with Cy3 fluorescent dye using Agilent one color low-input QuickAmp labeling kit following the manufacturer's instructions. Starting from 50 ng of total RNA purified from cell culture samples, amplification yield was 6.4 ± 0.9 µg cRNA and specific activity was 14.4 ± 1.0 pmol Cy3 per µg cRNA. The same amount of Cy3-labeled cRNA (600 ng) was fragmented at 60°C for 30 min in a reaction volume of 25 µl containing 1× Agilent fragmentation buffer and 2× Agilent blocking agent, according to the manufacturer's instructions. At the end of the reaction, 25 µl of 2× Agilent hybridization buffer (HI-RPM) was added to the fragmentation mixture. Forty µL of the mixture were then hybridized to Agilent SurePrint G3 human GE v3 8x60K microarrays for 17 h at 65°C in a rotating Agilent hybridization oven. After hybridization, microarrays were washed 1 min at room temperature with GE Wash Buffer 1 (Agilent) and 1 min with GE Wash buffer 2 at 37 °C (Agilent). Microarrays were scanned immediately after washing with the Agilent DNA Microarray C Scanner (G2565) using one color scan setting for 8x60k array slides. The scanned images were analyzed with Feature Extraction Software 10.7.3.1 (Agilent Technologies) using default parameters (protocol GE1_107_Sep09_ssSurrogates and Grid: 072363_D_F_20150612). After a standard quality control, all 36 microarrays (3 replicates per condition) from the genomic dataset were qualified and further analyzed using GeneSpring software (Agilent Technologies). Microarray data were first normalized based on the 75th percentile shift algorithm. Then, a filtration by "flag" and "signal intensity" was applied.

Were retained only the entities in which at least 100% of the values in any of the twelve conditions had a “detected flag” (i.e. a positive and significant feature as defined by GeneSpring). For the filtration by signal intensity, were retained the entities in which at least 100% of the values in any of the twelve conditions were within the range of interest (i.e. 20–100th percentile). Differentially expressed genes were identified by a three-way analysis of variance using GeneSpringGX software. Microarray data were submitted to the gene expression omnibus (GEO) database (www.ncbi.nlm.nih.gov/geo; GSE102536).

Evaluation of the mRNA expression of *crp* in the liver of zebrafish larvae

To assess the mRNA expression of *crp* in liver, liver samples of each larva were collected by laser microdissection capture prior to mRNA extraction. Briefly, after treatment, cryosections of larvae were realized to collect liver tissue under microscopic control on LMC system (Laser Microdissection Capture, Veritas, Life technologies [Carlsbad, CA]; platform H2P2, Biosit, Université de Rennes 1). For each sample of liver mRNA, approximatively 230000 μm^2 of tissue were collected and processed for mRNA extraction using PicoPure® RNA Isolation Kit (Thermo Fisher Scientific,Waltham, MA). From each mRNA, a retro-transcription step was performed on 20 to 50 ng using High capacity cDNA Reverse Transcription Kit (Applied Biosystems, Foster City, CA). Finally, Real-time quantitative PCR was performed using the fluorescent dye SYBR Green with the double-strand specific SYBR® Green system (Applied Biosystems) and the CFX384 Touch™ Real-Time PCR Detection System (Bio-Rad). cDNA (1 ng / well) was used as template for amplification with specific primer pairs (Table S1 - Online resource 1). Data of *crp* mRNA expression were normalized by *actb2* mRNA levels. Differential gene expression was calculated by the ΔΔCT calculation method.

REFERENCES

Coulouarn C, Corlu A, Glaise D, Guénon I, Thorgeirsson SS, Clément B (2012) Hepatocyte–stellate cell cross-talk in the liver engenders a permissive inflammatory microenvironment that drives progression in hepatocellular carcinoma. Cancer Res 72:2533–2542.

Supplementary Table S1: List of primers used for RT-qPCR experiments

Species	Gene	Accession Number	Forward primer	Reverse primer
Human	*APOA4*	NM_000104.3	tgatggacgcctttatcctc	ccacgacctgatccaattc
Human	*CYP1A1*	NM_000761.4	gacatctttggagcaggatttg	ccttctggatcttcctctgtatc
Human	*CYP1A2*	NM_000499.4	tcttccttcgtccccttcac	acaccttgtcgatagcacca
Human	*CYP1B1*	NM_003194.4	gagagttctgggattgtaccg	atcctcatgattaccgcagc
Human	*IL-6*	NM_000600.4	aaagaggcactggcagaaaa	ttcaccaggcaagtctcct
Human	*TBP*	NM_000482.3	cagtgtggcaagaaactcct	gtagtcccacatcaccgtg
Rat	Actb	NM_031144.3	gcaggagtacgatgagtccg	acgcagctcagtaacagttcc
Rat	*Crp*	NM_017096.3	gtccctggaagcagagtcaaa	ctcacatcagcgtgggcata
Rat	*Fgf-21*	NM _130752.1	cagatgcggtcgcttctttc	tctgcgccccatctgaattt
Rat	*Tnfα*	NM_012675.3	cgtcgtagcaaaccaccaa	tgaagagaacctgggagtagat
Zebrafish	*actb2*	NM_181601.4	ttctcttaagtcgacaacccc	taccaaccatgacaccctgat
Zebrafish	*18s*	NR_145818.1	ttaccccaggctcggaaaac	cgggaaggtctttgaaccca
Zebrafish	*gapdh*	NM_001115114.1	gaggcttctcacaaacgagga	tggccacgatctccactttc
Zebrafish	*apoa2*	NM_001130586.1	tcatcaaaatcgctggtggtt	caacatagggtctgaagtaatggc
Zebrafish	*cyp2y3*	NM_001020822.1	tattcccatgctgcactctg	aggagcgtttacctgcagaa
Zebrafish	*crp*	NM_001045860.1	cattagaggctaccgaaggttt	gactcaggggtttttcaggata
Zebrafish	*tnfa*	NM_212859.2	atttcacttccaaggctgcca	agctgatgtgcaaagacacc
Zebrafish	*il1b*	NM_212844.2	gaacagaatgaagcacatcaaacc	acggcactgaatccaccac

Supplementary Table S2: Phase I and II XME gene expression in HepaRG cells

		- FA						+ FA					
		- E			+ E			- E			+ E		
	GENE NAME	B 0	B 1	B 2.5	B 0	B 1	B 2.5	B 0	B 1	B 2.5	B 0	B 1	B 2.5
Phase I enzymes	*ADHFE1*	1.00	0.93	0.84	0.95	0.91	0.79	0.83	0.76	0.65	0.84	0.81	0.69
	ADH6	1.00	0.91	0.70	1.06	1.03	0.75	0.73	0.70	0.45	0.84	0.78	0.51
	ADH4	1.00	0.81	0.61	1.12	1.11	0.74	0.65	0.58	0.33	0.78	0.71	0.38
	ADH1C	1.00	0.82	0.64	1.11	1.01	0.69	0.58	0.50	0.31	0.68	0.60	0.37
	ADH1A	1.00	0.80	0.63	1.07	1.02	0.68	0.56	0.50	0.30	0.64	0.57	0.33
	ADH1B	1.00	0.78	0.56	1.04	0.93	0.60	0.43	0.39	0.24	0.49	0.41	0.23
	AKR7A2P1	1.00	0.94	0.81	1.02	0.96	0.80	0.72	0.73	0.61	0.78	0.74	0.65
	AKR7A3	1.00	0.90	0.74	1.03	0.95	0.73	0.67	0.64	0.52	0.72	0.67	0.54
	AKR7L	1.00	0.86	0.71	1.03	0.94	0.71	0.66	0.64	0.50	0.72	0.65	0.51
	AKR1C8P	1.00	0.79	0.77	0.88	0.84	0.66	0.59	0.49	0.43	0.52	0.43	0.38
	ALDH1A3	1.00	2.07	3.51	1.52	2.09	3.23	1.80	3.11	6.52	1.97	3.73	6.46
	ALDH3A1	1.00	2.36	5.66	1.06	1.99	4.44	0.86	2.09	5.02	1.00	2.04	5.08
	ALDH3B1	1.00	1.28	1.48	0.87	1.11	1.34	1.16	1.26	1.57	1.21	1.23	1.61
	ALDH1A1	1.00	0.91	0.91	1.04	0.88	0.84	0.75	0.69	0.67	0.74	0.72	0.66
	ALDH5A1	1.00	0.81	0.73	0.86	0.83	0.67	0.85	0.77	0.63	0.78	0.78	0.65
	ALDH1L2	1.00	0.85	0.86	1.15	1.25	0.90	1.57	1.28	0.99	1.33	1.38	0.64
	ALDH1L1	1.00	0.87	0.77	1.03	0.89	0.76	0.65	0.57	0.49	0.69	0.61	0.55
	ALDH6A1	1.00	0.95	0.80	0.93	0.91	0.72	0.75	0.74	0.55	0.79	0.70	0.52
	ALDH4A1	1.00	0.83	0.62	0.93	0.81	0.63	0.78	0.71	0.49	0.88	0.70	0.51
	ALDH8A1	1.00	0.71	0.51	0.95	0.73	0.55	0.93	0.56	0.40	0.89	0.60	0.40
	CYP1A1	1.00	8.65	47.53	1.03	3.10	20.84	0.95	5.62	40.69	0.74	4.14	24.40
	CYP1B1	1.00	3.86	12.84	0.95	2.52	6.53	1.07	3.42	13.21	1.07	2.96	9.00
	CYP1A2	1.00	3.33	10.97	0.91	1.54	4.91	0.54	1.63	4.67	0.54	1.09	3.12
	CYP19A1	1.00	1.77	3.27	0.81	1.32	2.41	1.30	1.98	3.65	1.47	1.77	2.86
	CYP2E1	1.00	0.64	0.36	0.91	0.74	0.41	1.62	1.14	0.59	1.73	1.32	0.76
	CYP4X1	1.00	0.70	0.53	1.01	0.92	0.65	0.96	0.76	0.58	1.10	1.03	0.70
	CYP4B1	1.00	0.79	0.75	1.08	0.86	0.84	0.90	0.62	0.55	1.06	0.73	0.66
	CYP4F12	1.00	0.96	0.89	1.02	1.01	0.87	0.72	0.68	0.63	0.76	0.68	0.65
	CYP4F8	1.00	0.88	0.81	1.01	0.93	0.77	0.75	0.66	0.56	0.74	0.70	0.59
	CYP3A5	1.00	0.79	0.62	0.90	0.77	0.57	0.87	0.72	0.56	0.89	0.74	0.59
	CYP4Z1	1.00	0.76	0.60	0.91	0.88	0.68	1.08	0.82	0.67	1.03	0.97	0.56
	CYP2B6	1.00	1.18	1.09	0.95	0.94	0.76	0.77	0.82	0.60	0.76	0.71	0.54
	CYP4A22	1.00	0.66	0.44	0.98	0.70	0.75	0.90	0.67	0.39	0.85	0.71	0.53
	CYP3A7	1.00	0.75	0.54	0.91	0.71	0.52	0.83	0.66	0.47	0.83	0.66	0.50
	CYP4F3	1.00	0.82	0.70	0.96	0.82	0.67	0.73	0.63	0.46	0.71	0.62	0.48
	CYP2C18	1.00	0.82	0.61	0.93	0.84	0.61	0.84	0.72	0.48	0.86	0.73	0.47
	CYP2A13	1.00	0.70	0.59	1.18	0.75	0.58	0.69	0.44	0.31	0.69	0.46	0.44
	CYP2C8	1.00	0.72	0.54	0.94	0.74	0.48	0.85	0.58	0.41	0.86	0.67	0.41
	CYP2C19	1.00	0.77	0.61	0.94	0.75	0.51	0.65	0.54	0.35	0.68	0.54	0.36
	CYP2C9	1.00	0.76	0.56	0.91	0.71	0.47	0.66	0.54	0.36	0.68	0.52	0.33
	CYP4A11	1.00	0.64	0.40	0.80	0.55	0.31	0.84	0.56	0.29	0.77	0.52	0.30
	CYP8B1	1.00	0.90	0.48	0.86	0.81	0.43	0.82	0.67	0.31	0.81	0.61	0.29
	CYP4F2	1.00	0.73	0.47	0.87	0.70	0.43	0.70	0.52	0.28	0.64	0.49	0.27
	CYP2A7	1.00	0.64	0.56	1.02	0.64	0.47	0.61	0.34	0.25	0.60	0.37	0.27
	CYP7A1	1.00	0.57	0.25	0.95	0.67	0.28	0.52	0.31	0.12	0.51	0.32	0.13
	CYP3A4	1.00	0.46	0.29	0.85	0.41	0.24	0.46	0.24	0.11	0.47	0.25	0.12
	EPHX2	1.00	0.81	0.62	0.99	0.89	0.63	0.76	0.70	0.48	0.82	0.71	0.50
Phase II enzymes	*GSTM2*	1.00	0.84	0.70	0.99	0.91	0.71	0.78	0.65	0.54	0.81	0.70	0.67
	GSTM4	1.00	0.89	0.74	1.03	0.90	0.74	0.76	0.69	0.56	0.83	0.71	0.67
	GSTM1	1.00	0.85	0.75	1.05	0.91	0.76	0.73	0.68	0.63	0.81	0.73	0.66
	GSTA5	1.00	0.92	0.81	1.03	0.95	0.80	0.69	0.65	0.52	0.74	0.68	0.56
	GSTA2	1.00	0.90	0.80	1.01	0.97	0.80	0.63	0.63	0.53	0.69	0.66	0.54
	GSTM2P1	1.00	0.91	0.68	1.45	1.09	0.94	1.36	0.80	0.65	1.22	0.98	0.51
	GSTA7P	1.00	0.88	0.91	0.77	0.70	0.62	0.55	0.52	0.50	0.43	0.40	0.41
	SULT2A1	1.00	0.76	0.69	0.98	0.87	0.76	0.81	0.67	0.57	0.90	0.76	0.63
	SULT1B1	1.00	0.91	0.76	0.93	0.93	0.63	1.03	0.97	0.69	1.03	0.91	0.56
	SULT1C2	1.00	0.91	0.74	0.91	0.96	0.70	0.80	0.74	0.50	0.80	0.72	0.46
	UGT2B7	1.00	0.98	0.90	0.87	0.88	0.81	0.71	0.75	0.69	0.72	0.73	0.66
	UGT2B11	1.00	0.82	0.70	0.93	0.84	0.67	0.85	0.75	0.64	0.78	0.77	0.66
	UGT2B15	1.00	0.80	0.71	0.93	0.78	0.66	0.76	0.68	0.61	0.75	0.72	0.65
	UGT2B10	1.00	0.85	0.70	0.97	0.85	0.66	0.83	0.74	0.62	0.81	0.76	0.61
	UGT2A3	1.00	0.85	0.83	0.91	0.77	0.78	0.67	0.67	0.63	0.62	0.64	0.59
	UGT2B4	1.00	0.80	0.56	1.03	0.82	0.57	0.90	0.75	0.55	0.92	0.79	0.58

Fold change

≥ 3

1

≤ 3

Supplementary Figure S1

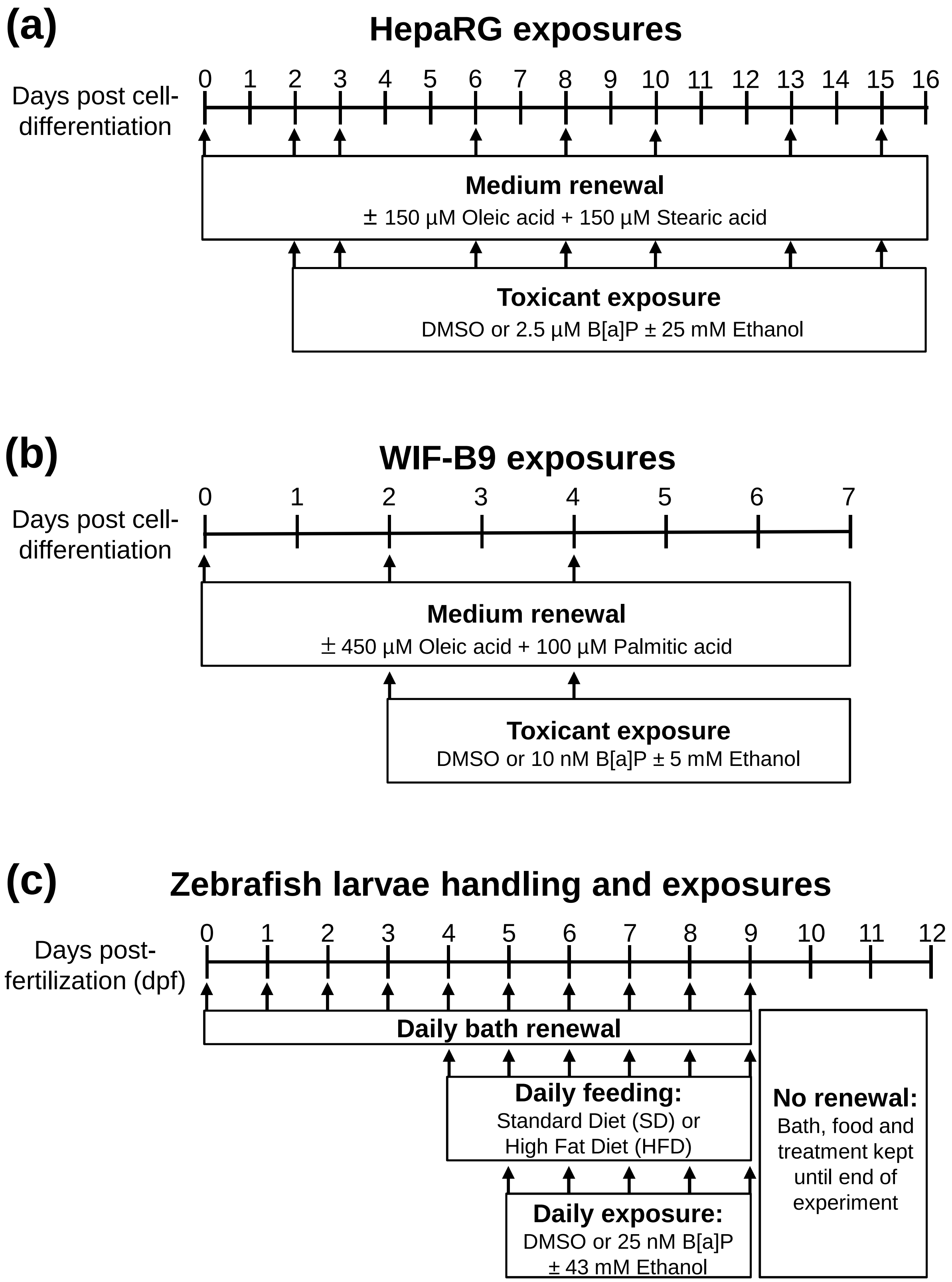

***In vitro* and *in vivo* protocols of exposure to toxicants**

Supplementary Figure S2

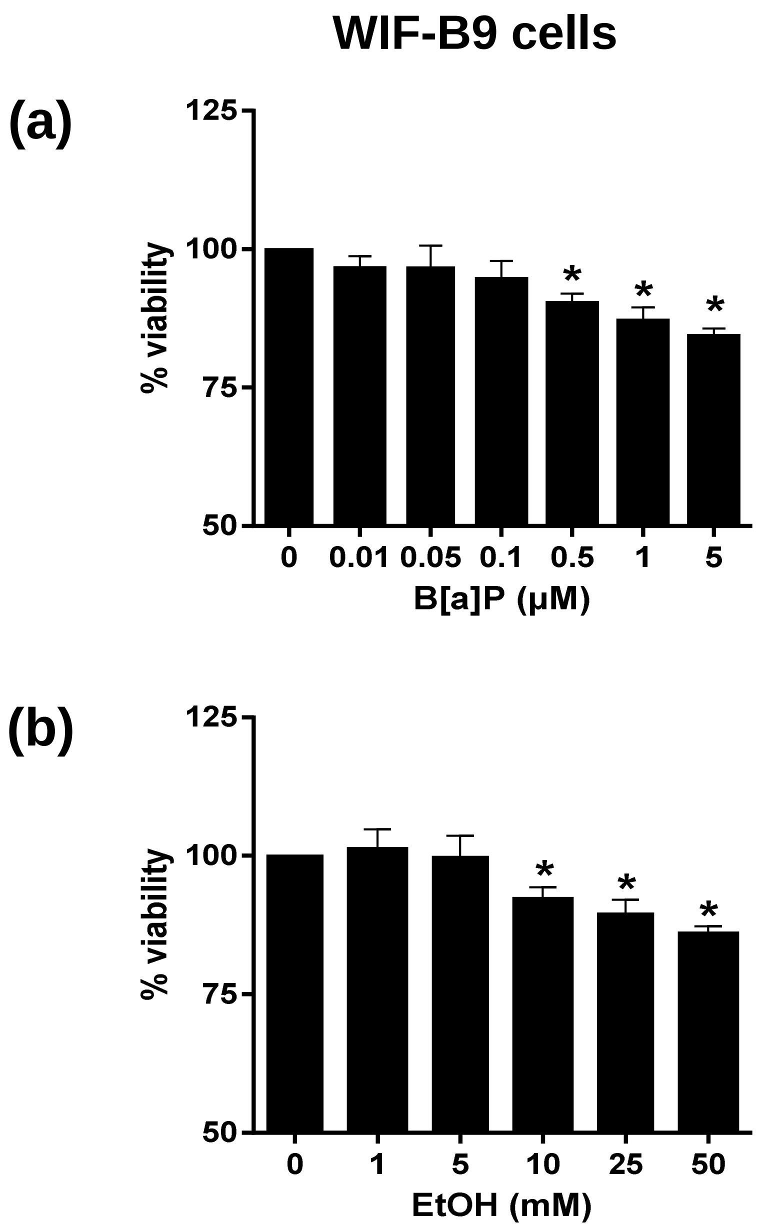

Dose-response cytotoxicity of benzo[a]pyrene and ethanol in non steatotic WIF-B9 cells. Cell viability was measured by the MTT (3-(4,5-dimethylthiazol-2-yl)-2,5-diphenyltetrazolium bromide) assay. Following 5 days of treatment with toxicants [from 0.01 to 5 µM for benzo[a]pyrene ***(a)***, and 1 to 50 µM for ethanol ***(b)***], cells were incubated for 2 hours with 0.5 mg/ml MTT solution. After removal of the MTT, formazan crystals were solubilized in DMSO and absorbance was directly measured using a Spectrostar Nano microplate reader (BMG Labtech). Values from 560 nm absorbance were subtracted from 670 nm absorbance to negate the background effect. Values are the mean ± SEM of at least three independent experiments. * Significantly different from untreated cells (t-test).

Supplementary Figure S3

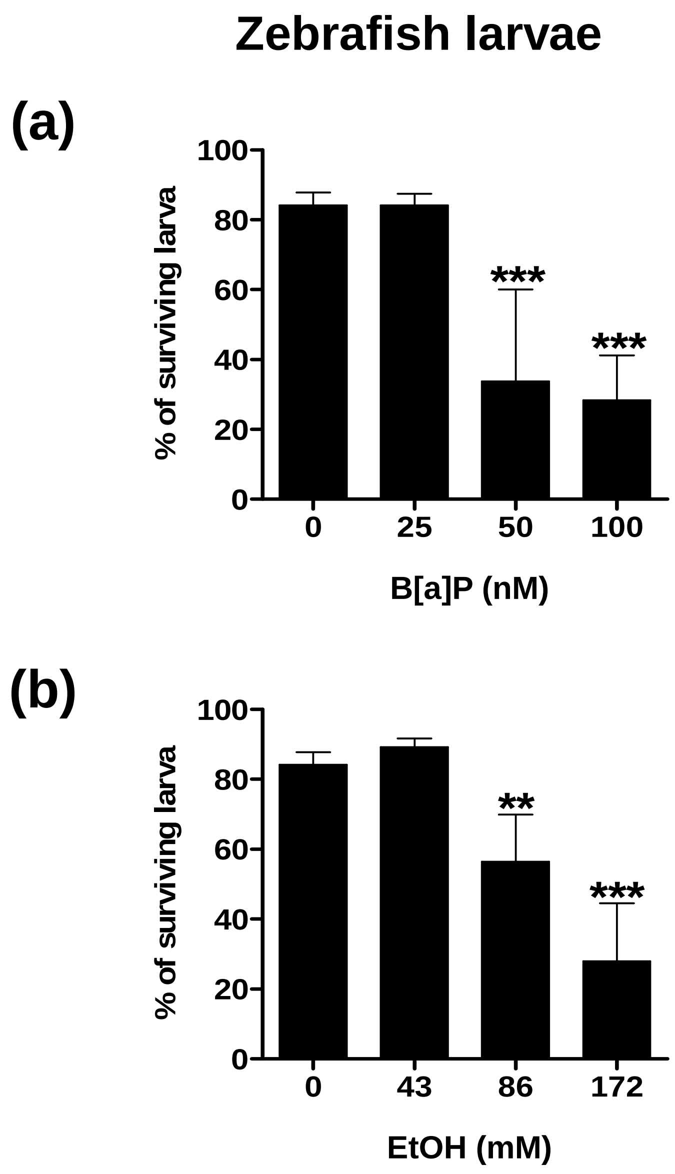

Dose-responses to B[a]P or ethanol on the survival rate of zebrafish larvae at 12 dpf. Zebrafish larvae were fed with a standard diet (SD) from 4 dpf until 12 dpf. From 5 dpf, SD larvae were treated or not for 7 days with different doses of ***(a)*** B[a]P or ***(b)*** ethanol. At the end of exposure (*i.e.* at 12 dpf), percentages of surviving larvae were calculated in comparison to the number of larvae included in each condition at 5 dpf. Values are the mean ± SEM of at least three batches of larvae. ** P<0.01; *** P<0.001 when compared to untreated larvae (one way analysis of variance followed by the Newman-Keuls multiple comparison test).

Supplementary Figure S4

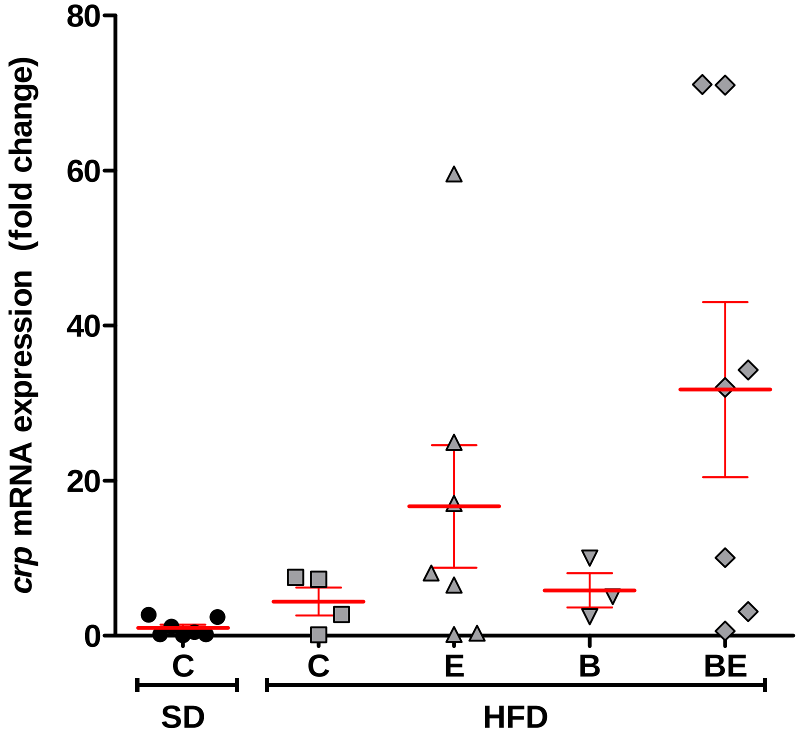

Induction of *crp* mRNA expression in liver of zebrafish larvae. Zebrafish larvae were fed with a standard diet (SD) or a high fat diet (HFD). HFD larvae were either left untreated (C), or treated with 25 nM B[a]P (B), 43 mM ethanol (E) or a combination of both toxicants (BE) for 7 days prior to mRNA extraction and analysis of *crp* mRNA expression (cf. supplementary Material and Methods – Online Resource 2). Values are the mean ± SEM of at least three independent experiments or larvae. Data are expressed relative to mRNA level found in untreated (C) SD larvae, arbitrarily set at 1 unit.

Supplementary Figure S5

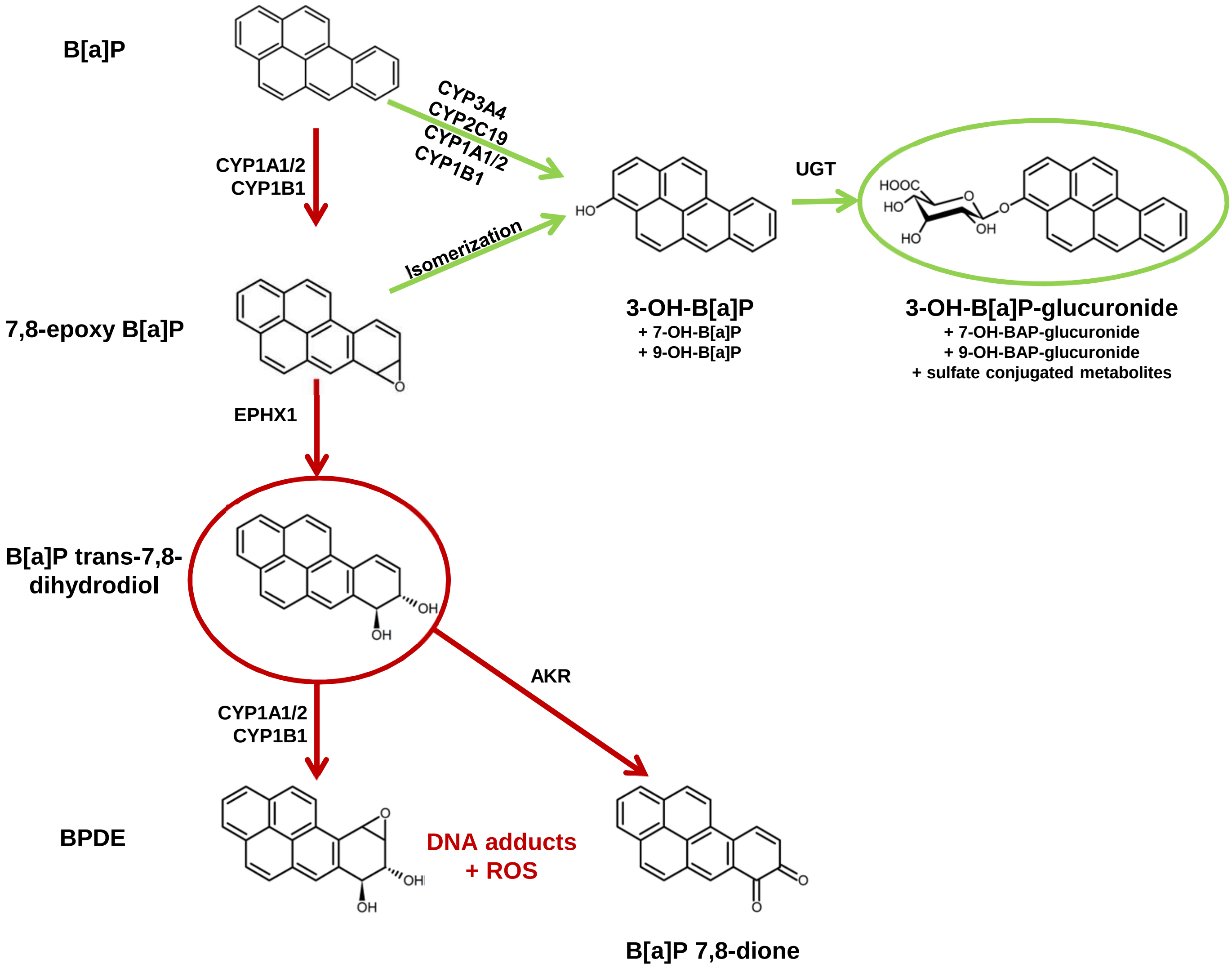

Schematic representation of B[a]P metabolism by phase I and II XMEs. Abbreviations: AKR, aldo-keto reductase; BPDE, (±)-anti-B[a]P-diol-epoxide; CYP, cytochrome P450; EPHX, epoxide hydrolase; ROS, reactive oxygen species; UGT, UDP-glucuronosyl transferase.

Supplementary Figure S6

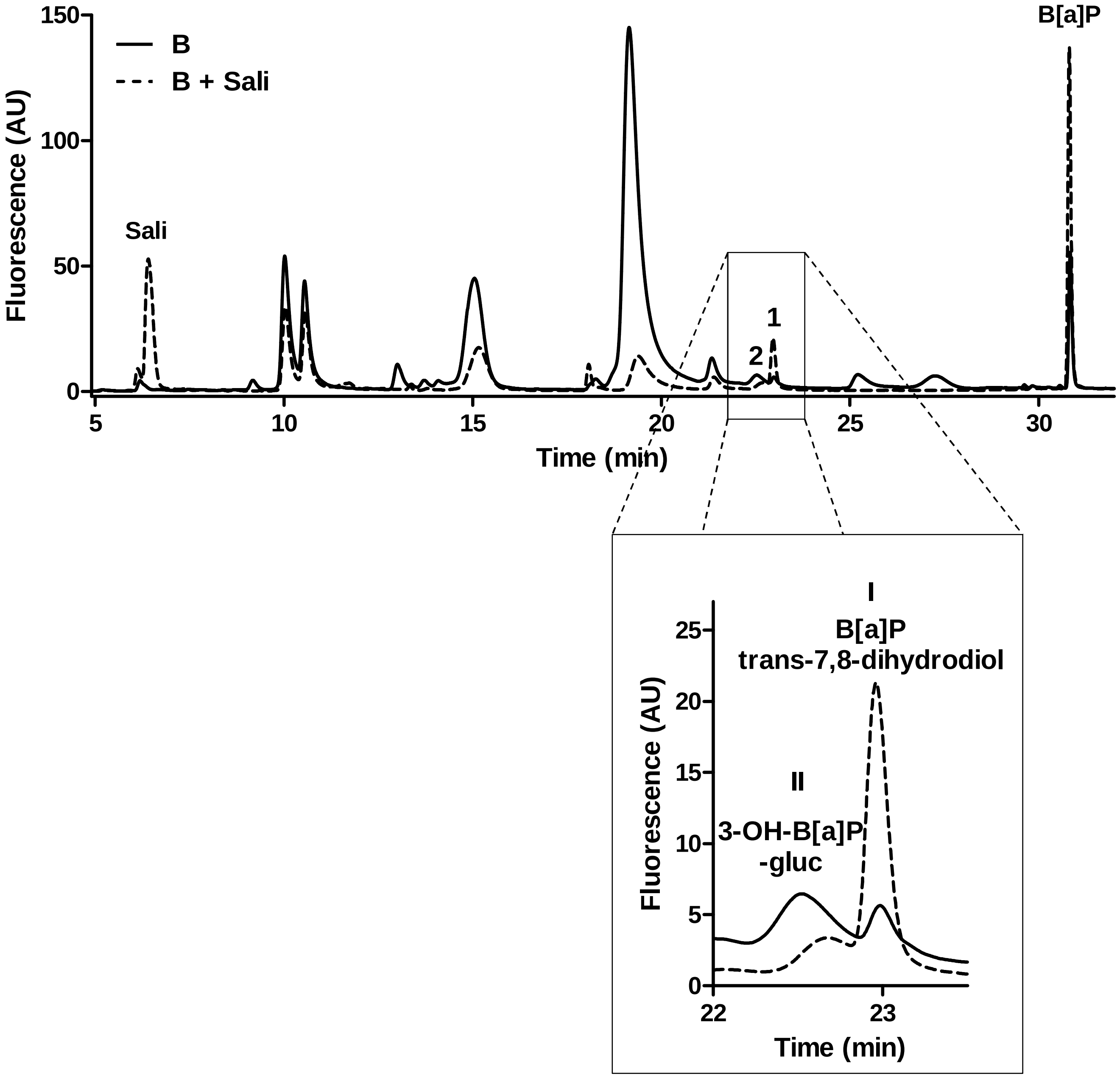

Effect of salicylamide on the production of phase I and II B[a]P metabolites. Non-steatotic HepaRG cells were treated for 14 days with 2.5 µM B[a]P. At the end of B[a]P exposure and after a 15-minute washout, B[a]P metabolites were analyzed in the culture media after an acute incubation of 25 µM B[a]P without (B) or with (B + Sali) 5 mM salicylamide, a strong inhibitor of phase II XMEs. One representative HPLC chromatogram was selected for each experimental condition. I and II indicate that the peaks correspond to phase I or phase II B[a]P metabolites, respectively. A magnification of the peaks corresponding to B[a]P trans-7,8-dihydrodiol and 3-OH-B[a]P-glucuronide is shown below the chromatograms. As expected, salicylamide treatment induced a reduction of the peak corresponding to 3-OH-B[a]P-glucuronide, and concomitant increase in the peak corresponding to B[a]P trans-7,8-dihydrodiol.
